# On asymptotic stability of solitons for 2D Maxwell–Lorentz equations with spinning particle

**DOI:** 10.1007/s00605-025-02064-3

**Published:** 2025-03-17

**Authors:** E. Kopylova

**Affiliations:** Faculty of Mathematics, Vienna University, Vienna, Austria

**Keywords:** Maxwell–Lorentz equations, Rotating particle, Solitons, Asymptotic stability, 35L70, 35Q61, 37K06, 37K40

## Abstract

We consider 2D Maxwell–Lorentz equations with an extended charged rotating particle. The system admits solitons which are solutions corresponding to a particle moving with constant velocity and rotating with constant angular velocity. Our main result is asymptotic stability of moving solitons with zero angular velocity.

## 2D Maxwell–Lorentz equations with spinning particle

The 2D Maxwell–Lorentz system reads

1.1 $$\begin{aligned} \left\{ \begin{array}{rcl} \dot{E}(x,t)& =& J\nabla B(x,t)-[\dot{q}(t)-\omega (t) J(x-q(t))] \rho (x-q(t))\\ \dot{B}(x,t)& =& -\nabla \cdot (JE(x,t)), \qquad \nabla \cdot E(x,t)=\rho (x-q(t))\\ m\ddot{q}(t)& =& \langle E(x,t)+B(x,t) [J\dot{q}(t) +\omega (t) (x-q(t)) ],\rho (x\!-\!q(t))\rangle \\ I\dot{\omega }(t)& =& \langle (x\!-\!q(t))\cdot \big [J E(x,t)\!-B(x,t) \dot{q}(t)],\rho (x\!-\!q(t))\rangle \end{array}\right| ,~~x\in \mathbb {R}^2,~~t\in \mathbb {R}. \end{aligned}$$

Here $E(x,t), q(t)\in \mathbb {R}^2$, $B(x,t), \omega (t)\in \mathbb {R}$, $$J=\begin{pmatrix} 0&  1\\ -1&  0\end{pmatrix}$$, and the brackets $\langle \cdot ,\cdot \rangle$ denote the inner product in the Hilbert space $L^2:=L^2(\mathbb {R}^2)\otimes \mathbb {R}^2$. The system describes a motion of an extended charged particle, centered at q(t), in the Maxwell field (*E*(*x*, *t*), *B*(*x*, *t*)). Moreover, $m>0$ is the mass of the particle and $I>0$ is its moment of inertia. Note that the system is an analogue of 3D Maxwell–Lorentz system [8, (2.39)–(2.41)].

We assume that the charge density $\rho (x)$ is real-valued, compactly supported and spherically symmetric, i.e.,

1.2 $$\begin{aligned} \rho \in C_0^\infty (\mathbb {R}^2),\quad \rho (x)=0 ~~\textrm{for}~~|x|\ge R_{\rho },\quad \rho (x)=\rho _{rad}(|x|),\quad \rho (x)\not \equiv 0. \end{aligned}$$

Moreover, we assume that

1.3 $$\begin{aligned} \int \rho (x) dx=0,\qquad \int x^2\rho (x) dx=0. \end{aligned}$$

Equivalently, $\hat{\rho }(k)=\mathcal{O}(|k|^4)$ as $k\rightarrow 0$. In terms of the Maxwell potentials $\Phi (x,t)$ and $A(x,t)=(A_1(x,t),A_2(x,t))$, we have

1.4 $$\begin{aligned} B(x,t)=\nabla \cdot (JA),\qquad E(x,t)=-\dot{A}(x,t)-\nabla \Phi (x,t). \end{aligned}$$

We choose the Coulomb gauge $\nabla \cdot A(x,t)=0$. Then the second line of (1.6) implies

$$ -\Delta \Phi (x,t)=\rho (x-q(t)). $$

Hence, $\Phi (x,t)=\Phi _0(x-q(t))$, where $\Phi _0(x)=-\displaystyle \frac{1}{2\pi }\int \log (|x-y|)\rho (y)dy$. Denote

1.5 $$\begin{aligned} p(t)=m\dot{q}+\langle A(x,t),\rho (x-q(t))\rangle ,\quad M(t)=I\omega -\langle A(x,t),J\varrho (x-q(t))\rangle , \quad \varrho (x)=x\rho (x) \end{aligned}$$

By (1.1) and (1.4),

$$\begin{aligned} \dot{p}= &   m\ddot{q}+\langle \dot{A}(x),\rho (x-q)\rangle -\langle A(x),(\dot{q}\cdot \nabla )\rho (x-q)\rangle \\= &   \langle -\dot{A}(x)+(\nabla \cdot JA(x)) [J\dot{q} +\omega (x-q)],\rho (x-q)\rangle \\  &   +\langle \dot{A}(x),\rho (x-q)\rangle -\langle 
A(x),(\dot{q}\cdot \nabla )\rho (y)\rangle \\= &   -\langle A(x)\cdot \dot{q},\nabla \rho (x-q)\rangle +\omega \langle \nabla \cdot JA(x), \varrho (x-q)\rangle ,\\ \dot{M}= &   I\dot{\omega }-\langle \dot{A}(y+q),J\varrho (y)\rangle -\langle (\nabla \cdot \dot{q})A(y+q),J\varrho (y)\rangle \\= &   -\langle (\nabla \cdot JA(y+q))\dot{q},\varrho (y)\rangle -\langle (\nabla \cdot \dot{q})A(y+q),J\varrho (y)\rangle \\= &   -\langle A(y+q)\cdot \dot{q}, \nabla \cdot J\varrho (y)\rangle =0 \end{aligned}$$

Hence, in the Maxwell potentials, system (1.1) reads

1.6 $$\begin{aligned} \left\{ \!\begin{array}{llll} \dot{A}(x,t)=\Pi (x,t) \\ \dot{\Pi }(x,t)=\Delta A(x,t)-\omega (t) J\varrho (x-q(t))+\mathcal{P}[\dot{q}(t)\rho (x-q(t))]\\ m\dot{q}(t)=p-\langle A(x,t),\rho (x-q(t))\rangle \\ \dot{p}(t)=-\langle A(x,t)\cdot \dot{q}(t),\nabla \rho (x-q(t))\rangle +\omega (t) \langle \nabla \cdot JA(x,t), \varrho (x-q(t))\rangle \end{array}\!\right| , \end{aligned}$$

where $\omega (t)=\frac{1}{I}\big (M+\!\langle A(x,t),J\varrho (x-q(t))\rangle \big )$, and $\mathcal{P}$ is the projection onto the space of solenoidal (divergence-free) vector fields. In the Fourier space

1.7 $$\begin{aligned} \widehat{\mathcal{P}f}(k)=\hat{f}(k)-\frac{\hat{f}(k)\cdot k}{k^2}k=\frac{\hat{f}(k)\cdot Jk}{k^2}Jk. \end{aligned}$$

The system admits *soliton* solutions (3.1) that move with constant velocities $v\in \mathbb {R}^2$, $0\le |v|<1$ and rotate with constant angular velocities $\textrm{w}\in \mathbb {R}$. Asymptotic stability of the soliton with velocity $v_0$ and angular velocitiy $\textrm{w}_0$ means that for solutions with initial data sufficiently close to this soliton the following properties are satisfied:

(i) $$\dot{q}(t)\rightarrow v_\pm \approx v_0$$ and $$\omega (t)\rightarrow \omega _\pm \approx \textrm{w}_0$$ as $$t\rightarrow \pm \infty $$;
(ii) for the field part of the solutions asymptotics (4.2) hold, where the remainder $r_{\pm }(x,t)\rightarrow 0$ as $t\rightarrow \pm \infty$ in the global energy norm.

Our main results are as follows. We prove asymptotic stability of solitons

1. with $v_0\ne 0$ and $\textrm{w}=0$ in the case of large $I\gg 1$ (Theorem 4.1).
2. with small $|v_0|\ll 1$ and $\textrm{w}=0$ under the Wiener condition
  1.8 $$\begin{aligned} \hat{\rho }(k)\ne 0~~\textrm{for}~~ k\ne 0 \end{aligned}$$
  (Theorem 4.2).

Our results extend the result of [7], where the system without rotation was considered (the first three lines of (1.1) with $\omega =0$).

## Hamiltonian structure and well-posedness

Denote by $L^2_{\beta }(\mathbb {R}^2)$ the weighted Sobolev space with the norm

$$ \Vert f\Vert _{L^2_{\beta }(\mathbb {R}^2)}=\Vert (1+|x|)^{\beta }f\Vert _{L^2(\mathbb {R}^2)}. $$

Denote by $\dot{H}^1_\beta$ the closure of $C_0^\infty (\mathbb {R}^2)\otimes \mathbb {R}^2$ with respect to the norm $\Vert f\Vert _{\dot{H}^1_\beta }=\Vert \nabla f\Vert _{L^2_\beta }$, where $L^2:=L^2(\mathbb {R}^2)\otimes \mathbb {R}^2$. Let $\textbf{L}^2_\beta$, $\dot{\textbf{H}}^1_\beta$ be the subspaces of $L^2_\beta$ and $\dot{H}^1_\beta$ formed by solenoidal vector fields. We define the space

$$ \mathcal{E}_{\beta }:=\dot{\textbf{H}}^1_\beta \oplus \textbf{L}^2_\beta \oplus \mathbb {R}^2\oplus \mathbb {R}^2 $$

of the states $Y=(A,\Pi ,q,p)$ with the norm

$$ \Vert Y\Vert _{\beta }=\Vert \nabla A\Vert _{\textbf{L}^2_{\beta }}+\Vert \Pi \Vert _{\textbf{L}^2_{\beta }}+|q|+|p|. $$

Besides, we will use the space $\mathcal{F}_{\beta }:=\dot{\textbf{H}}^1_\beta \oplus \textbf{L}^2_\beta$ of the solenoidal vector fields $(A,\Pi )$ with the norm $\Vert (A,\Pi )\Vert _{\mathcal{F}_\beta }=\Vert \nabla A\Vert _{\textbf{L}^2_{\beta }}+\Vert \Pi \Vert _{\textbf{L}^2_{\beta }}$.

Of course, the case $\beta =0$ corresponds to the usual $L^2$, $\dot{H}^1$, $\mathcal{E}$ and $\mathcal{F}$ spaces without weight.

System (1.6) is a Hamiltonian system with the Hamiltonian functional

2.1 $$\begin{aligned} H(Y)=\frac{1}{2}\int [|\Pi (x)|^2+|\nabla A(x)|^2]dx+\frac{1}{2}m\dot{q}^2+\frac{1}{2} I\omega ^2,~~ Y=(A,\Pi , q, p)\in \mathcal{E} \end{aligned}$$

where $$\dot{q}=\frac{1}{m}(p-\langle A(x),\rho (x-q)\rangle )$$, $$I=\frac{1}{\omega }(M+\!\langle A(x),J\varrho (x\!-\!q)\rangle )$$.

### Proposition 2.1

Let (1.2) holds, and let $Y_0=(A_0, \Pi _0, q_0, p_0)\in \mathcal{E}$, $M\in \mathbb {R}$. Then

(i) there exists a unique solution $Y(t)\in C(\mathbb {R}, \mathcal{E})$ to the Cauchy problem for (1.6);
(ii) the energy conserves: $H(Y(t))=H(Y_0)$ for $t\in R$;

The proof is similar to [3, Lemma A.2].

## Solitons and solitary manifold

Solitons of the system (1.6) are solutions of the form

3.1 $$\begin{aligned} Y_{v}(x,t)=(A_{v}(x-vt-a),\Pi _{v}(x-vt-a), vt+a, p_{v}),~~\textrm{where}~~~p_{v}=mv+\langle A_{v},\rho \rangle . \end{aligned}$$

Substituting (3.1) into the first two equations of (1.6), we get

3.2 $$\begin{aligned} \Pi _{v}(y)=-(v\cdot \nabla )A_{v}(y), \qquad -(v\cdot \nabla )\Pi _{v}(y)=\Delta A_{v}(y)-\textrm{w}J\varrho (y)+\mathcal{P}[v\rho (y)], \end{aligned}$$

where $\textrm{w}=\textrm{w}(v)=\frac{1}{I}\big (M+\langle A_{v},J\varrho \rangle )$. Hence, $\Delta A_{v}(y)-(v\cdot \nabla )^2 A_{v}(y)=\textrm{w}J\varrho (y)-\mathcal{P}_s[v\rho (y)]$. This equation is easy to solve in the case $|v|<1$. Namely, in the Fourier representation, we get

3.3 $$\begin{aligned} \hat{A}_{v}(k)= \frac{(v-\frac{(v\cdot k)k}{k^2})\hat{\rho }(k)}{\hat{D}_0}-\frac{\textrm{w}J\hat{\varrho }(k)}{\hat{D}_0},~~\textrm{where}~~~\hat{D}_0:=k^2-(v\cdot k)^2. \end{aligned}$$

The third and the fourth equations of (1.6) for $Y_{v}$ are easily verified. By (3.3),

3.4 $$\begin{aligned} \langle A_{v},J\varrho \rangle =-\textrm{w}\left\langle \frac{J\hat{\varrho }(k)}{\hat{D}_0},J\hat{\varrho }(k)\right\rangle =-\textrm{w}\varkappa _0, \quad \varkappa _0=\int \frac{|\hat{\varrho }(k)|^2}{\hat{D}_0}dk=\int \frac{|\nabla \hat{\rho }(k)|^2}{\hat{D}_0}dk. \end{aligned}$$

Hence, $\textrm{w}=0$ for $M=0$. In this case, conditions (1.2)–(1.3) imply that

3.5 $$\begin{aligned} \nabla _j A_{v},~ \Pi _{v}\in L^2_{\beta }, \qquad j=1,2,\quad \beta <4 \end{aligned}$$

(see [7, Lemma 3.1]). Denote $V:=\{v\in \mathbb {R}^2:|v|<1\}$.

### Definition 3.1

$S(\sigma )a:=(A_{v}(x-b),\Pi _{v}(x-b),b, p_{v})$, where $\sigma :=(b,v)$ with $b\in \mathbb {R}^2$ and $v\in V$.

A solitary manifold is the set $\mathcal{S}:=\{S(\sigma ):\sigma \in \Sigma :=\mathbb {R}^2\times V\}$.

Now we define “symplectic orthogonal projection” onto $\mathcal{S}$. Denote

$$ \mathcal{E}^+=\{Y=(A,\Pi , p,q)\in \mathcal{E}: \hat{\Pi }(k)/|k|\in L^2\}. $$

Denote the symplectic form $\Omega$

3.6 $$\begin{aligned} \Omega (Y_1,Y_2)=\langle Y_1,\textbf{J}Y_2\rangle ,\quad Y_1\in \mathcal{E}, \quad Y_2\in \mathcal{E}^+,\quad \textbf{J}:=\left( \begin{array}{ccccc} 0 &  I_2 &  0 &  0 \\ -I_2 &  0 &  0 &  0\\ 0 &  0 &  0 &  I_2 \\ 0 &  0 &  -I_2 &  0 \end{array}\right) . \end{aligned}$$

where $$ \langle Y_1, Y_2\rangle :=\langle A_1, A_2\rangle + \langle \Pi _1,\Pi _2\rangle +q_1 q_2+p_1 p_2. $$

### Definition 3.2

$Y_1\not \mid Y_2$ means that $Y_1$ is symplectic orthogonal to $Y_2$, i.e. $\Omega (Y_1,Y_2)=0$.

Denote by $\mathcal{T}_{\sigma }\mathcal{S}$ to the tangent space to the manifold $\mathcal{S}$ at a point $S(\sigma )$. The vectors

3.7 $$\begin{aligned} \left. \begin{array}{rclrclrclrclrclrcl} \tau _j(v):=\partial _{b_j}S(\sigma )=& (-\partial _j A_{v}(y),& -\partial _j\Pi _{v}(y),& e_j&  0) \\ \tau _{j+2}(v):=\partial _{v_j}S(\sigma )=& (\partial _{v_j}A_{v}(y),& \partial _{v_j}\Pi _{v}(y),&  0,&  \partial _{v_j}p_{v}) \end{array}\right| ,~ j=1,2, \end{aligned}$$

form a basis in $\mathcal{T}_{\sigma }\mathcal{S}$. Here $e_1=(1,0)$, $e_2=(0,1)$.

### Lemma 3.3

[7, Lemma 5.2] The matrix $\mathbf{\Omega }=\mathbf{\Omega }(v)$ with the elements $\Omega (\tau _l(v),\tau _j(v))$ is non-degenerate for any $v\in V$.

The next lemma states that in a small neighborhood of the solitary manifold $\mathcal{S}$ a “symplectic orthogonal projection” onto $\mathcal{S}$ is well-defined. Denote $\mathcal{E}_\alpha (\overline{v})=\{Y(A,\Pi ,q,p)\in \mathcal{E}_\alpha : |v(Y)|\le \overline{v}\}$, where $v(Y)=\frac{1}{m}(p-\langle A(\cdot ),\rho (\cdot -y)\rangle )$.

### Lemma 3.4

Let (1.2) hold, $\beta \in \mathbb {R}$ and $\overline{v}<1$. Then

(i) there exists a neighborhood $\mathcal{O}_\beta (\mathcal{S})$ of $\mathcal{S}$ in $\mathcal{E}_\beta$ and a map $\mathbf{\Pi }:\mathcal{O}_\beta (\mathcal{S})\rightarrow \mathcal{S}$ such that $\mathbf{\Pi }$ is uniformly continuous on $\mathcal{O}_\beta (\mathcal{S})\cap \{Y\in \mathcal{E}_\beta : |v(Y)|\le {\overline{v}}\}$ in the metric of $\mathcal{E}_\beta$,
  3.8 $$\begin{aligned} \mathbf{\Pi } Y=Y~~\text{ for }~~ Y\in \mathcal{S}, ~~~~~\text{ and }~~~~~Y-S \not \mid \mathcal{T}_S\mathcal{S},~~\text{ where }~~S=\mathbf{\Pi } Y. \end{aligned}$$
(ii) For any $\overline{v}<1$ there exists a $\tilde{v}<1$ s.t. $|v(\mathbf{\Pi }Y)|<\tilde{v}$ when $|v(Y)|<\overline{v}$.
(iii) For any $\tilde{v}<1$ there exists a $0<z_\beta (\tilde{v})<1$ s.t. $S(\sigma )+Z\in \mathcal{O}_\beta (\mathcal{S})$ if $|v(S(\sigma ))|<\tilde{v}$ and $\Vert Z\Vert _\beta <z_\beta (\tilde{v})$.

The proof is similar to that of Lemma 3.4 in [2]. We will call $\mathbf{\Pi }$ the symplectic orthogonal projection onto $\mathcal{S}$.

## Main results

### Theorem 4.1

Let conditions (1.2)–(1.3) hold, $I\gg 1$ and $7/2<\beta <4$. Suppose that $Y_0\in \mathcal{E}_{\beta }$ such that $M(Y_0)=0$, and

4.1 $$\begin{aligned} Y_0=S(v_0, b_0)+Z_0~~\textrm{with}~~0< |v_0|<1,\quad \textrm{and}~~d_\beta =\Vert Z_0\Vert _{\beta }\ll 1. \end{aligned}$$

Let $Y(t)\in C(\mathbb {R}, \mathcal{E})$ be the solution to (1.6) with initial data $Y_0$. Then

4.2 $$\begin{aligned}  &   \dot{q}(t)= v_{\pm }+\mathcal{O}(|t|^{-2}),\quad q(t)=v_{\pm }t+a_{\pm }+\mathcal{O}(|t|^{-2}), \quad \omega _{\pm }(t)=\mathcal{O}(|t|^{-2}),\quad t\rightarrow \pm \infty , \end{aligned}$$

4.3 $$\begin{aligned}  &   \left( \begin{array}{cc} A(x,t)\\ \Pi (x,t)\end{array}\right) =\left( \begin{array}{cc} A_{v_{\pm }}(x-v_{\pm }t-a_{\pm })\\ \Pi _{v_{\pm }}(x-v_{\pm }t-a_{\pm })\end{array}\right) +W_0(t)\Psi _{\pm }+r_{\pm }(x,t),\quad t\rightarrow \pm \infty . \end{aligned}$$

Here $W_0(t)$ is the dynamical group of the free wave equation, $\Psi _{\pm }$ are the corresponding asymptotic scattering states, and the remainder $r_{\pm }(x,t)$ converges to zero in the global energy norm:

4.4 $$\begin{aligned} \Vert r_{\pm }(t)\Vert _\mathcal{F}=\mathcal{O}(|t|^{-1}),\quad t\rightarrow \pm \infty . \end{aligned}$$

### Theorem 4.2

Let conditions (1.2)–(1.3) and Wiener condition (1.8) hold, and $7/2<\beta <4$. Suppose that $Y_0\in \mathcal{E}_{\beta }$ such that $M(Y_0)=0$, and

4.5 $$\begin{aligned} Y_0=S(v_0, b_0)+Z_0~~\textrm{with}~~|v_0|\ll 1,\quad \textrm{and}~~d_\beta =\Vert Z_0\Vert _{\beta }\ll 1. \end{aligned}$$

Let $Y(t)\in C(\mathbb {R}, \mathcal{E})$ be the solution to (1.6) with initial data $Y_0$. Then asymptotics (4.2)–(4.4) hold.

## Linearization on the solitary manifold

We split a solution *Y*(*t*) to the system (1.6) as the sum $Y(t)=S(\sigma (t))+Z(t)$, where $Z=(\Lambda ,\Psi ,r,\pi )$, and $\sigma (t)=(b(t),v(t))\in \Sigma$ is an arbitrary smooth function of $t\in \mathbb {R}$. In detail,

5.1 $$\begin{aligned} \left. \begin{array}{rclr} A(x,t)& =& A_{v(t)}(x-b(t))+\Lambda (x-b(t),t), ~~~\Pi (x,t)=\Pi _{v(t)}(x-b(t))+\Psi (x-b(t),t)\\ q(t)& =& b(t)+r(t),~~~~~~~~~~~~~~~~~~~~~~~~~~~~~~p(t)=p_{v(t)}+\pi (t) \end{array} \right| \end{aligned}$$

Substituting (5.1) to (1.6) and setting $y=x-b(t)$, we obtain

$$ \left\{ \begin{array}{llll} \dot{A}& =& \dot{v}\cdot \nabla _v A_{v(t)}(y)-\dot{b}\cdot \nabla A_{v(t)}(y)+ \dot{\Lambda }(y,t)-\dot{b}\cdot \nabla \Lambda (y,t)=\Pi _{v(t)}(y)+\Psi (y,t)\\ \dot{\Pi }& =& \dot{v}\cdot \nabla _v\Pi _{v(t)}(y)-\dot{b} \cdot \nabla \Pi _{v(t)}(y)+\dot{\Psi }(y,t)-\dot{b}\cdot \nabla \Psi (y,t)=\Delta A_{v(t)}(y)+\Delta \Lambda (y,t)\\ & & -\frac{1}{I}\langle A_{v(t)}(y)+\Lambda (y,t),J\varrho (y-r)\rangle J\varrho (y-r)+\mathcal{P}[\dot{q} \rho (y-r)]\\ m\dot{q}& =& m(\dot{b}+\dot{r})=p_{v}+\pi -\langle A_{v(t)}+\Lambda ,\rho (y-r)\rangle \\ \dot{p}& =& \dot{v}\cdot \nabla _v p_{v(t)}+\dot{\pi }(t) =-\langle (A_{v(t)}(y)+\Lambda (y,t))\cdot \dot{q},\nabla \rho (y-r)\rangle \\ & +& \frac{1}{I}\langle A_{v(t)}(y)+\Lambda (y,t),J\varrho (y-r)\rangle \langle \nabla \cdot J(A_{v(t)}(y)+\Lambda (y,t)),\varrho (y-r)\rangle \end{array}\right. $$

Using (3.2), we obtain the system for *Z*(*t*):

5.2 $$\begin{aligned} \left\{ \begin{array}{llll} \!\!\dot{\Lambda }(y,t)\!\!\!& =& \!\!\Psi (y,t)+\dot{b}\cdot \nabla \Lambda (y,t)+ (\dot{b}-v)\cdot \nabla A_{v}(y)-\dot{v}\cdot \nabla _v A_{v}(y)\\ \!\!\dot{\Psi }(y,t)\!\!\!& =& \!\!\Delta \Lambda (y,t)+\dot{b}\cdot \!\nabla \Psi (y,t)-\dot{v}\cdot \!\nabla _v \Pi _{v}(y) +(\dot{b}\!-\!v)\cdot \! \nabla \Pi _{v}(y)+\mathcal{P}[\dot{q} \rho (y\!-\!r)-v\rho (y)]\\ \!\!& -& \!\!\frac{1}{I}\big (\langle A_{v}(y)+\Lambda (y,t),J\varrho (y\!-\!r)\rangle J\varrho (y\!-\!r) -\langle A_{v},J\varrho \rangle \big ) J\varrho (y)\big )\\ ~~~m\dot{r}\!\!\!& =& \!\!-m\dot{b}+mv+\langle A_v,\rho \rangle +\pi -\langle A_v(y)+\Lambda (y,t),\rho (y-r)\rangle \\ ~~~~\dot{\pi }\!\!\!\!& =& \!\!-\dot{v}\cdot \nabla _v p_{v}-\langle (A_v(y)+\Lambda (y,t))\cdot \dot{q},\nabla \rho (y-r)\rangle \\ \!\!& +& \!\!\frac{1}{I}\langle A_v(y)+\Lambda (y,t),J\varrho (y-r)\rangle \langle \nabla \cdot J(A_v(y)+\Lambda (y,t),\varrho (y-r)\rangle \end{array}\!\right| \end{aligned}$$

Denote the symmetric matrix *P* and *Q* with the entries

5.3 $$\begin{aligned} P_{lj}= \int \frac{k_l\nabla _j\hat{\rho }(k)}{\hat{D}_0}\hat{\rho }(k) dk,\quad Q_{jl}=\int \big (v^2k^2-(v\cdot k)^2\big )\frac{k_lk_j|\hat{\rho }(k)|^2dk}{k^2\hat{D}_0}, \quad j,l=1,2. \end{aligned}$$

Simple calculation based on (3.3) gives

5.4 $$\begin{aligned}  &   \langle A_{v} r\cdot \nabla J\varrho \rangle =-\left\langle \frac{(v_1k_2^2-v_2k_1k_2)\hat{\rho }}{k^2\hat{D}_0}, (r\cdot k)\nabla _2\hat{\rho }\right\rangle +\left\langle \frac{(v_2k_1^2-v_1k_1k_2)\hat{\rho }}{k^2\hat{D}_0}, (r\cdot k)\nabla _1\hat{\rho }\right\rangle \nonumber \\  &   \qquad =-\left\langle \frac{\big ((v_1k_2-v_2k_1)k_2^2-(v_2k_1-v_1k_2)k_1^2\big )\hat{\rho }}{k^2\hat{D}_0}, r\cdot \nabla \hat{\rho }\right\rangle \nonumber \\  &   \qquad =\left\langle \frac{(v_2k_1-v_1k_2)\hat{\rho }}{\hat{D}_0}, r\cdot \nabla \hat{\rho }\right\rangle =r\cdot PJv, \end{aligned}$$

5.5 $$\begin{aligned}  &   \langle \nabla \cdot JA_{v},\varrho \rangle =\int \frac{(v_2k_1-v_1k_2)\hat{\rho }}{\hat{D}_0}\,\nabla \hat{\rho }dk=PJ v, \end{aligned}$$

5.6 $$\begin{aligned}  &   \langle A_{v}\cdot v, \nabla (r\cdot \nabla \rho )\rangle =-\int \frac{v^2k^2-(v\cdot k)^2}{k^2\hat{D}_0}k(r\cdot k)|\hat{\rho }(k)|^2dk=-Qr. \end{aligned}$$

Now we will extract linear terms in the RHS of (5.2). First, note that

5.7 $$\begin{aligned} \rho (y-r)=\rho (y)-r \cdot \nabla \rho (y)+N_{\rho }(r,y), \end{aligned}$$

where $N_{\rho }(r,y)=\rho (y-r)-\rho (y)+r \cdot \nabla \rho (y)$. The expansion and the third equation of (5.2) imply

5.8 $$\begin{aligned} \dot{r}=-\dot{b}+v+\frac{1}{m}\big (\langle A_v,\rho \rangle +\pi -\langle A_v+\Lambda ,\rho (y-r)\rangle \big ) =-\dot{b}+v+\frac{1}{m}\big (\pi -\langle \Lambda ,\rho \rangle \big )+N_3, \end{aligned}$$

where

5.9 $$\begin{aligned} \Vert N_3(\sigma ,Z)\Vert _{\beta }\le C(\tilde{v})\Vert Z\Vert _{-\beta }^2, \end{aligned}$$

uniformly for $\Vert Z\Vert _{-\beta }\le z_{-\beta }(\tilde{v})$ and $|v|<\tilde{v}<1$. Further, (5.1) and (5.7)–(5.8) imply

5.10 $$\begin{aligned} \dot{q}\rho (y-r)-v\rho (y) =\frac{\rho (y)}{m} \big (\pi -\langle \Lambda ,\rho \rangle )-vr \cdot \nabla \rho (y)+N_2'. \end{aligned}$$

Similarly, (5.4) and (5.7) imply

5.11 $$\begin{aligned} \langle A_v+\Lambda ,J\varrho (y-r)\rangle J\varrho (y-r)-\langle A_v,J\varrho \rangle J\varrho =-(r\cdot PJv)J\varrho +\langle \Lambda ,J\varrho \rangle J\varrho +N_2''. \end{aligned}$$

Substituting (5.10) and (5.11) into the second equation of (5.2), we obtain

5.12 $$\begin{aligned} \dot{\Psi }= &   \Delta \Lambda +\dot{b}\cdot \!\nabla \Psi \!+(\dot{b}\!-\!v)\!\cdot \! \nabla \Pi _{v}\! -\dot{v}\cdot \nabla _v \Pi _{v}+\mathcal{P}\Big [\frac{\rho }{m}\big (\pi -\langle \Lambda ,\rho \rangle \big )-vr \cdot \nabla \rho \Big ]\\ \nonumber  &   +\frac{1}{I}\big (r\cdot PJv-\langle \Lambda ,J\varrho \rangle \big )J\varrho +N_2 \end{aligned}$$

where for $N_2=N_2(\sigma , Z)$ the bound of type (5.9) holds. Further, using (5.6) and (5.7), we get

5.13 $$\begin{aligned} \langle (A_v+\Lambda )\cdot \dot{q},\nabla \rho (y-r)\rangle -\langle A_v\cdot v,\nabla \rho \rangle =\langle \Lambda \cdot v,\nabla \rho \rangle +Qr+N_4'. \end{aligned}$$

Moreover, (5.4), (5.5) and (5.7) imply

5.14 $$\begin{aligned}  &   \frac{1}{I}\langle A_v+\Lambda ,J\varrho (y-r)\rangle \langle \nabla \cdot J(A_v+\Lambda ),\varrho (y-r)\rangle -\frac{1}{I}\langle A_v,J\varrho \rangle \langle \nabla \cdot JA_v,\varrho \rangle \nonumber \\  &   \qquad =\frac{1}{I}\langle \Lambda ,J\varrho \rangle PJv -\frac{1}{I}(r\cdot PJv) PJv+N_4''. \end{aligned}$$

Substituting (5.13) and (5.14) into the fourth equation of (5.2), we obtain

5.15 $$\begin{aligned} \dot{\pi }=-\dot{v}\cdot \nabla _v p_{v}-\langle v\cdot \Lambda ,\nabla \rho \rangle -Qr +\frac{1}{I}\langle \Lambda ,J\varrho \rangle PJv-\frac{1}{I}(r\cdot PJv) PJv+N_4, \end{aligned}$$

where for $N_4=N_4(\sigma , Z)$ the bound of type (5.9) holds.

Denote $u(t)=\dot{b}(t)$. Combining the first equation of (5.2) together with equations (5.12), (5.8), and (5.15), we obtain

5.16 $$\begin{aligned} \dot{Z}(t)=\textbf{A}_{u(t), v(t)}Z(t)+T_{u(t), v(t)}+N(\sigma (t),Z(t)),\,\,\,t\in \mathbb {R}, \quad Z=(\Lambda ,\Psi ,r,\pi ), \end{aligned}$$

where

5.17 $$\begin{aligned}  &   \textbf{A}_{u,v}:=\left( \begin{array}{cccccc} u \cdot \nabla &  1 &  0 &  0 \\ \Delta -\mathcal{P}\left[ \frac{\langle \cdot ,\rho \rangle \rho }{m}\right] -\frac{\langle \cdot ,J\varrho \rangle J\varrho }{I}&  u \cdot \nabla &  -\mathcal{P}[v(\cdot \nabla \rho )]+(\cdot PJv)\frac{J\varrho }{I} & \mathcal{P}[\frac{\rho }{m}] \\ -\frac{1}{m}\langle \cdot ,\rho \rangle &  0 &  0&  \frac{1}{m}\\ -\langle v\cdot ,\nabla \rho \rangle +\frac{1}{I}PJv\langle \cdot ,J\varrho \rangle &  0 & -Q-\frac{(\cdot PJv) PJv}{I}&  0\\ \end{array}\right) , \end{aligned}$$

5.18 $$\begin{aligned}  &   T_{u, v}:=\left( \begin{array}{c} (u-v)\cdot \nabla A_{v}-\dot{v}\cdot \nabla _v A_{v}\\ (u-v)\!\cdot \nabla \Pi _{v}-\dot{v}\cdot \nabla _v \Pi _{v}\\ v-u \\ -\dot{v}\cdot \nabla _v p_{v}\\ \end{array} \right) ,\quad N(\sigma ,Z):=\left( \begin{array}{c} 0 \\ N_2(\sigma ,Z) \\ \ N_3(\sigma ,Z) \\ N_4 (\sigma ,Z) \end{array} \right) . \end{aligned}$$

The remainder term $N(\sigma ,Z)$ satisfies the estimate

5.19 $$\begin{aligned} \Vert N(\sigma ,Z)\Vert _\beta \le C(\tilde{v})\Vert Z\Vert ^2_{-\beta } \end{aligned}$$

uniformly in *v* and *Z* with $\Vert Z\Vert _{-\beta }\le z_{-\beta }(\tilde{v})$ and $|v|<\tilde{v}<1$.

## The linearized equation

### Lemma 6.1

The operator $\textbf{A}_{u,v}$ acts on the tangent vectors $\tau _j(v)$ to the solitary manifold as follows:

6.1 $$\begin{aligned} \textbf{A}_{u,v}[\tau _j(v)]=(u-v)\cdot \nabla \tau _j(v),\quad \textbf{A}_{u,v}[\tau _{j+2}(v)]=(u-v)\cdot \nabla \tau _{j+2}(v)+\tau _j(v,),\qquad j=1,2. \end{aligned}$$

### Proof

Differentiating equations (3.2) in $b_j$ and $v_j$, we obtain:

$$\begin{aligned} \!\!\!\!\!\!\!\!-(v\cdot \nabla )\partial _j A_{v}= &   \partial _j\Pi _{v},\qquad -(v\cdot \nabla )\partial _j\Pi _{v}=\Delta \partial _j A_{v}+\mathcal{P}[v\partial _j\rho ],\\ \!\!\!\!\!\!\!\!-\partial _j A_{v}-(v\cdot \nabla )\partial _{v_j} A_{v}= &   \partial _{v_j}\Pi _{v},\qquad -\partial _j\Pi _{v}- (v\cdot \nabla )\partial _{v_j}\Pi _{v}=\Delta \partial _{v_j} A_{v}+\mathcal{P}[e_j\rho ]. \end{aligned}$$

Moreover, by (3.1), $\partial _{v_j}p_{v}= me_j+\langle \partial _{v_j}A_{v},\rho \rangle$. Then (6.1) follows by the definition (5.17) of $\textbf{A}_{v,u}$.

Denote $$\textbf{A}_{v}:=\textbf{A}_{v,v}$$.

### Corollary 6.2

The tangent vectors $\tau _j(v)$ are eigenvectors, and $\tau _{j+2}(v)$ are root vectors of $\textbf{A}_{v}$, corresponding to zero eigenvalue, i.e. $\textbf{A}_{v}[\tau _j(v)]=0$, $\textbf{A}_{v}[\tau _{j+2}(v)]=\tau _j(v)$, $j=1,2$;

Now we study some Hamiltonian properties of the linear equation

6.2 $$\begin{aligned} \dot{X}(t)=\textbf{A}_{v}X(t),\quad t\in \mathbb {R},\quad v\in V. \end{aligned}$$

### Lemma 6.3

(i) Equation (6.2) formally can be written as
  6.3 $$\begin{aligned} \dot{X}(t)=\textbf{J}\mathcal{D}\mathcal{H}_{v}(X(t)),\quad X=(\Lambda ,\Psi ,r,\pi )\in \mathcal{E},\quad t\in \mathbb {R}, \end{aligned}$$
  where $\mathcal{D}\mathcal{H}_{v}$ is the Fréchet derivative of the Hamilton functional
  6.4 $$\begin{aligned} \mathcal{H}_{v}(X)= &   \frac{1}{2}\int \big (|\Psi |^2+|\nabla \Lambda |^2\big ) dy+\int \Psi (v\cdot \nabla )\Lambda dy +\frac{1}{2m}(\pi -\langle \Lambda ,\rho \rangle )^2\nonumber \\  &   +\frac{1}{2I}\big (r\cdot PJv+\langle \Lambda ,J\varrho \rangle \big )^2+\int (\nabla \rho \cdot r)v\cdot \Lambda dy+\frac{r\cdot Qr}{2}. \end{aligned}$$
(ii) $\mathcal{H}_{v}(X(t))=\textrm{const}$, $t\in \mathbb {R}$, for solutions $X(t)\in C^1(\mathbb {R},\mathcal{E})$.
(iii) The Hamilton function $\mathcal{H}_{v}(X)$ is nonnegative definite,
  6.5 $$\begin{aligned} \mathcal{H}_{v}(X)\ge 0,\quad X\in \mathcal{E}. \end{aligned}$$

### Proof

Equality (6.3) is easily verified by differentiation. The energy conservation law follows by (6.3) and the chain rule for the Fréchet derivatives since the operator $\textbf{J}$ is skew-symmetric. To prove iii), we notice that

$$ \int \big (|\Psi |^2+|\nabla \Lambda |^2\big ) dy+2\int \Psi (v\cdot \nabla )\Lambda dy=\Vert \Psi +(v\cdot \nabla )\Lambda \Vert ^2_{L^2(\mathbb {R}^2)} +\langle (-\Delta +(v\cdot \nabla )^2\Lambda ,\Lambda \rangle . $$

Hence, it remains to prove that

6.6 $$\begin{aligned} \langle (-\Delta +(v\cdot \nabla )^2\Lambda ,\Lambda \rangle +2\!\int (\nabla \rho \cdot r)v\cdot \Lambda dy + r\cdot Qr\ge 0. \end{aligned}$$

We can assume, without loss of generality, that $v=(|v|, 0)$. In this case, the matrix *Q* is diagonal, with the entries $q_j:=q_{jj}=v^2\displaystyle \int \frac{k_2^2k_j^2|\hat{\rho }(k)|^2\,dk}{k^2\hat{D}_0}$ by (5.3). Now, in the Fourier space, the LHS of (6.6) reads

$$\begin{aligned}  &   \int \big (\hat{D}_0|\hat{\Lambda }|^2+2(k\cdot r)\hat{\rho }|v|\hat{\Lambda }_1\big )\,dk+\sum \limits _{j}q_jr_j^2 =\int \Big (\hat{D}_0|\hat{\Lambda }|^2+2(k\cdot r)\hat{\rho }|v| \left( e_1-\frac{k_1k}{k^2}\right) \cdot \hat{\Lambda }\\  &   \quad +v^2\frac{(k\cdot r)^2|\hat{\rho }|^2}{\hat{D}_0}(e_1-\frac{k_1k}{k^2})^2\Big )\,dk =\int \Big (\Big [\hat{D}_0^{1/2}\hat{\Lambda }+\frac{(k\cdot r)|v|\hat{\rho }}{\hat{D}_0^{1/2}}(e_1-\frac{k_1k}{k^2})\Big ]^2\Big )\,dk. \end{aligned}$$

Here we used that $k\cdot \hat{\Lambda }=0$.

## Solution of the linearized equation

We change variables in Eq. (6.2) to simplify its structure. Denote

7.1 $$\begin{aligned} \phi :=\frac{1}{m}\big (\pi -\langle \Lambda ,\rho \rangle \big ),\quad \nu :=\frac{1}{I}\big (\langle \Lambda , J\varrho \rangle -r\cdot PJv\big ). \end{aligned}$$

Then for $\mathcal{X}=(\Lambda , \Psi , r, \phi )$, we obtain the following equation

7.2 $$\begin{aligned} \dot{\mathcal{X}}\!=\mathcal{A}_{v}\mathcal{X}=\left( \!\begin{array}{l} \Psi +v\cdot \nabla \Lambda \\ \Delta \Lambda +v\cdot \nabla \Psi + \mathcal{P}[\rho \phi -\!v(r\cdot \nabla \rho )]-\nu J\varrho \\ \phi \\ -\frac{1}{m}\Big (\langle v\cdot \Lambda ,\nabla \rho \rangle -\nu PJv+Qr +\langle \Psi +v\cdot \nabla \Lambda ,\rho \rangle \Big ) \end{array}\!\!\!\right) \end{aligned}$$

Applying the Laplace transform $\tilde{\mathcal{X}}(\lambda )=\int _0^\infty e^{-\lambda t}\mathcal{X}(t)dt$, $~\mathrm{Re{\hspace{0.5mm}}}\lambda >0$, we get

7.3 $$\begin{aligned} \left\{ \! \begin{array}{l} \tilde{\Psi }+v\cdot \nabla \tilde{\Lambda }-\lambda \tilde{\Lambda }=-\Lambda _0\\ \Delta \tilde{\Lambda }+v\cdot \!\nabla \tilde{\Psi }+\mathcal{P}\big [\big (\tilde{\phi }-v(\tilde{r}\cdot \nabla )\big )\rho \big ] -\tilde{\nu }J\varrho -\lambda \tilde{\Psi }=-\Psi _0\\ \tilde{\phi }-\lambda \tilde{r}=-r_0\\ \langle v\cdot \tilde{\Lambda },\nabla \rho \rangle +Q\tilde{r}-\tilde{\nu }PJv +\langle \tilde{\Psi }+v\cdot \nabla \tilde{\Lambda },\rho \rangle +m\lambda \tilde{\phi }=m\phi _0, \end{array} \right. \end{aligned}$$

We rewrite this system as

7.4 $$\begin{aligned} \left\{ \! \begin{array}{l} \tilde{\Psi }+v\cdot \nabla \tilde{\Lambda }-\lambda \tilde{\Lambda }=-\Lambda _0\\ \Delta \tilde{\Lambda }+v\cdot \!\nabla \tilde{\Psi }+\mathcal{P}\big [\big (\lambda \tilde{r}-v(\tilde{r}\cdot \nabla )\big )\rho \big ] -\tilde{\nu }J\varrho -\lambda \tilde{\Psi }=-\Psi _0+\mathcal{P}[r_0\rho ]\\ \tilde{\phi }=\lambda \tilde{r}-r_0\\ \langle v\cdot \tilde{\Lambda },\nabla \rho \rangle +m\lambda ^2\tilde{r}+Q\tilde{r}-\tilde{\nu }PJv+\lambda \langle \tilde{\Lambda },\rho \rangle =m\phi _0+\langle \Lambda _0,\rho \rangle +m\lambda r_0 \end{array} \right. \end{aligned}$$

*Step i)* The first two equations in Fourier space become

7.5 $$\begin{aligned}  &   \hat{\tilde{\Psi }}(k)-i(v\cdot k)\hat{\tilde{\Lambda }}(k)-\lambda \hat{\tilde{\Lambda }}(k)=-\hat{\Lambda }_0(k)\nonumber \\  &   -k^2\hat{\tilde{\Lambda }}(k)-i(v\cdot k)\hat{\tilde{\Psi }}(k)-\lambda \hat{\tilde{\Psi }}(k)=-\hat{\Psi }_0(k)+\widehat{\mathcal{P}[r_0\rho ]}(k)-\hat{\tilde{K}}(k) \end{aligned}$$

Here

7.6 $$\begin{aligned} \hat{\tilde{K}}(k)=\lambda \widehat{\mathcal{P}[\tilde{r}\rho ]}(k)+i(k\cdot \tilde{r})\widehat{\mathcal{P} [v\rho ]}(k)-\tilde{\nu }J\hat{\varrho }. \end{aligned}$$

One has

$$ \left( \begin{array}{cc} -(i(v\cdot k)+\lambda ) &  1 \\ -k^2 &  -(i(v\cdot k)+\lambda ) \end{array} \right) ^{-1}=\frac{1}{\hat{D}(\lambda )}\left( \begin{array}{cc} -(i(v\cdot k)+\lambda ) &  -1 \\ k^2 &  -(i(v\cdot k)+\lambda ) \end{array}\right) , $$

where $\hat{D}(\lambda )=\hat{D}(v,k,\lambda ):=(i(v\cdot k)+\lambda )^2+k^2$ does not vanish for $\mathrm{Re{\hspace{0.5mm}}}\lambda >0$ and $|v|<1$. Hence,

7.7 $$\begin{aligned} \hat{\tilde{\Lambda }} =\frac{\hat{K}_0}{\hat{D}(\lambda )}+\frac{\hat{\tilde{K}}}{\hat{D}(\lambda )},\quad \textrm{where}~~ \hat{K}_0:=(i(v\cdot k)+\lambda )\hat{\Lambda }_0+\hat{\Psi }_0-\widehat{\mathcal{P}[r_0\rho ]}. \end{aligned}$$

Substituting (7.7) into the forth equation of (7.4), we get

7.8 $$\begin{aligned}  &   i\left\langle \frac{v\cdot \hat{\tilde{K}}}{\hat{D}(\lambda )},k\hat{\rho }\right\rangle +\big (Q+m\lambda ^2\big )\tilde{r} -\tilde{\nu }PJv+\lambda \left\langle \frac{\hat{\tilde{K}}}{\hat{D}(\lambda )},\hat{\rho }\right\rangle \nonumber \\  &   \qquad =\pi _0+m\lambda r_0-i\left\langle \frac{v\cdot \hat{K}_{0}}{\hat{D}(\lambda )},k\hat{\rho }\right\rangle -\lambda \left\langle \frac{\hat{K}_0}{\hat{D}(\lambda )},\hat{\rho }\right\rangle . \end{aligned}$$

We will denote by $P_{lj}(\lambda )$, $Q_{lj}(\lambda )$ the function defined in (5.3) with $\hat{D}(\lambda )$ instead of $\hat{D}_0$. Due to (1.7) and (7.6),

7.9 $$\begin{aligned} \left\langle \frac{\hat{\tilde{K}}}{\hat{D}(\lambda )},\hat{\rho }\right\rangle =\lambda \left\langle \frac{\widehat{\mathcal{P}[\tilde{r}\rho ]}}{\hat{D}(\lambda )},\hat{\rho }\right\rangle =\lambda \int \frac{|\hat{\rho }|^2(\tilde{r}\cdot Jk)}{k^2\hat{D}(\lambda )} Jk\, dk=\lambda U(\lambda )\tilde{r}, \end{aligned}$$

where

7.10 $$\begin{aligned} U(\lambda )=\begin{bmatrix} u_{22}(\lambda )& -u_{12}(\lambda )\\ -u_{21}(\lambda )&  u_{11}(\lambda )\end{bmatrix} ~~\textrm{with}~~~u_{jl}(\lambda )=\int \frac{k_jk_l|\hat{\rho }|^2dk}{k^2\hat{D}(\lambda )}. \end{aligned}$$

Further, (1.7) and (7.6) and imply

7.11 $$\begin{aligned} \left\langle \frac{v\cdot \hat{\tilde{K}}}{\hat{D}(\lambda )},k\hat{\rho }\right\rangle =i\left\langle \frac{v^2k^2-(v\cdot k)^2}{k^2\hat{D}(\lambda )}\hat{\rho }(k\cdot \tilde{r}), k\hat{\rho }\right\rangle +i\tilde{\nu }\left\langle v\cdot \frac{J\nabla \hat{\rho }}{\hat{D}(\lambda )},k\hat{\rho }\right\rangle =iQ(\lambda )\tilde{r}-i\tilde{\nu }P(\lambda )Jv \end{aligned}$$

(cf. (5.5) and (5.6)). Substituting (7.9) and (7.11) into the LHS of (7.8), we obtain

7.12 $$\begin{aligned} \Big (\breve{Q}+\lambda ^2 U(\lambda )+m\lambda ^2\Big )\tilde{r}-\tilde{\nu }\breve{P}Jv= \pi _0+m\lambda r_0-i\left\langle \frac{v\cdot \hat{K}_{0}}{\hat{D}(\lambda )},k\hat{\rho }\right\rangle -\lambda \left\langle \frac{\hat{K}_0}{\hat{D}(\lambda )},\hat{\rho }\right\rangle , \end{aligned}$$

where $\breve{Q}=\breve{Q}(\lambda ):=Q-Q(\lambda )$, $\breve{P}:=P-P(\lambda )$. Let us express $\tilde{\nu }$ in terms of $\tilde{r}$. By (1.7) and (7.6),

7.13 $$\begin{aligned} \left\langle \frac{\hat{\tilde{K}}}{\hat{D}(\lambda )}, J\hat{\varrho }\right\rangle= &   -\left\langle \frac{(k\tilde{r})\hat{\rho }}{\hat{D}(\lambda )}v, J\nabla \hat{\rho }\right\rangle +\left\langle \frac{ (vk)(k\tilde{r})\hat{\rho }}{k^2\hat{D}(\lambda )}k, J\nabla 
\hat{\rho }\right\rangle -\tilde{\nu }\left\langle \frac{\nabla \hat{\rho }}{\hat{D}},\nabla \hat{\rho }\right\rangle \nonumber \\= &   \tilde{r}\cdot P(\lambda )Jv-\tilde{\nu }\varkappa (\lambda ),\qquad \varkappa (\lambda ):=\int \frac{|\nabla \hat{\rho }|^2}{\hat{D}(\lambda )}dk. \end{aligned}$$

Hence (7.1) and (7.7) imply that $I\tilde{\nu }=-\tilde{r}\cdot \breve{P}(\lambda )Jv-\tilde{\nu }\varkappa (\lambda )+\langle \frac{\hat{K}_0}{\hat{D}(\lambda )},J\hat{\varrho }\rangle$. Therefore,

7.14 $$\begin{aligned} \tilde{\nu }=\frac{1}{\kappa (\lambda )}\left( -\tilde{r}\cdot \breve{P}(\lambda )Jv+\left\langle \frac{\hat{K}_0}{\hat{D}(\lambda )},J\hat{\varrho }\right\rangle \right) , \quad \kappa (\lambda ):=I+\varkappa (\lambda ). \end{aligned}$$

Evidently, $\kappa (0)>0$. Moreover, $\kappa (\lambda )\ne 0$ for $\mathrm{Re{\hspace{0.5mm}}}\lambda >0$. In Appendix B we will show that $\mathrm{Im{\hspace{0.5mm}}}\kappa (i\mu +0)=\mathrm{Im{\hspace{0.5mm}}}\varkappa (i\mu +0)\ne 0$ for $\mu \in \mathbb {R}$. Substituting (7.14) into (7.12), we get

7.15 $$\begin{aligned} \Big (\breve{Q}(\lambda )+\lambda ^2 U(\lambda )+m\lambda ^2\Big )\tilde{r}+\frac{\tilde{r}\cdot \breve{P}(\lambda )Jv}{\kappa (\lambda )}\breve{P}(\lambda )Jv=\Gamma _0(\lambda ), \end{aligned}$$

where we denote

7.16 $$\begin{aligned} \Gamma _0(\lambda ):=\pi _0+m\lambda r_0-i\left\langle \frac{v\cdot \hat{K}_{0}}{\hat{D}(\lambda )},k\hat{\rho }\right\rangle -\lambda \left\langle \frac{\hat{K}_0}{\hat{D}(\lambda )},\hat{\rho }\right\rangle +\frac{1}{\kappa (\lambda )}\left\langle \frac{\hat{K}_0}{\hat{D}(\lambda )},J\hat{\varrho }\right\rangle \breve{P}(\lambda )Jv. \end{aligned}$$

Now we assume that $v=(|v|,0)$. Denote

7.17 $$\begin{aligned} p_j(\lambda )=\!\int \frac{\hat{\rho }(k)k_j\nabla _j\hat{\rho }(k)dk,}{\hat{D}(\lambda )}, \quad q_{j}(\lambda )=v^2\!\int \frac{k_2^2k_j^2|\hat{\rho }(k)|^2dk}{k^2\hat{D}(\lambda )},\quad u_j(\lambda )=\!\int \frac{(k^2-k_j^2)|\hat{\rho }|^2dk}{k^2\hat{D}(\lambda )}. \end{aligned}$$

Then

7.18 $$\begin{aligned} U=\begin{bmatrix} u_{1}&  0\\ 0&  u_{2}\end{bmatrix},\quad Q=\begin{bmatrix} q_{1} &  0\\ 0 & q_{2}\end{bmatrix}, \quad P=\begin{bmatrix} p_{1} &  0\\ 0 & p_{2}\end{bmatrix},\quad PJ v=-|v|p_2 e_2. \end{aligned}$$

Now (7.15) reads

7.19 $$\begin{aligned} \tilde{r}=L^{-1}(\lambda )\Gamma _0(\lambda ),\quad \textrm{where} \quad L(\lambda )=\begin{bmatrix} a_1(\lambda ) &  0\\ 0&  a_2(\lambda )+\frac{v^2(p_2(0)-p_2(\lambda ))^2}{\kappa (\lambda )}\end{bmatrix}. \end{aligned}$$

Here we denote

7.20 $$\begin{aligned} a_j(\lambda ):=q_j(0)-q_j(\lambda )+\lambda ^2(m+u_j(\lambda )). \end{aligned}$$

The invertibility of the matrix $L(\lambda )$ for $\mathrm{Re{\hspace{0.5mm}}}\lambda >0$ follows from the next lemma.

### Lemma 7.1

The operator $\mathcal{A}_v-\lambda :\mathcal{E}\rightarrow \mathcal{E}$ has a bounded inverse operator for $\mathrm{Re{\hspace{0.5mm}}}\lambda >0$.

### Proof

First, let us prove that Ker$\,(\mathcal{A}_v-\lambda )=0$ for $\mathrm{Re{\hspace{0.5mm}}}\lambda >0$. Let $\tilde{\mathcal{X}}_{\lambda }=\big (\tilde{\Lambda }_{\lambda },\tilde{\Psi }_{\lambda },\tilde{r}_{\lambda },\tilde{\phi }_{\lambda }\big )\in \mathcal{E}$ be a solution to (7.3) with $\mathcal{X}_0=0$. Then $X(t)=(\tilde{\Lambda }_{\lambda },\,\tilde{\Psi }_{\lambda },\,\tilde{r}_{\lambda },\,m\tilde{\phi }_{\lambda }+\langle \tilde{\Lambda }_{\lambda },\rho \rangle )e^{\lambda t}\in C[0,\infty ;\mathcal{E})$ is the solution to (6.2). Suppose that $\tilde{\phi }_{\lambda }\ne 0$. Then $\mathcal{H}_{v}(X(t))$ grows exponentially by (6.4) and (6.5), which contradicts Lemma 6.3-ii). Thus, $\tilde{\phi }_{\lambda }= 0$. Hence, $\tilde{r}_\lambda =0$ by the third equation of (7.3). Finally, $\tilde{\Lambda }_{\lambda }=0$ and $\tilde{\Psi }_{\lambda }=0$ by (7.7) and the first equation of (7.3), respectively.

By (7.3), $\mathcal{A}_v-\lambda =\mathcal{K}(\lambda )+\mathcal{T}$, where $$ \mathcal{K}(\lambda )=\begin{pmatrix} v\cdot \nabla -\lambda &  ~~1 & 0\\ ~~~\Delta &  v\cdot \nabla -\lambda & 0\\ ~~~0 &  0&  -\lambda I_4 \end{pmatrix}, $$ and $\mathcal{T}$ is finite-dimensional operator. The operator $\mathcal{K}^{-1}(\lambda )$ is bounded in $\mathcal{E}$ for $\mathrm{Re{\hspace{0.5mm}}}\lambda > 0$ (see [4, 6]). Moreover, $\mathcal{A}_v-\lambda =\mathcal{K}(I+\mathcal{K}^{-1}\mathcal{T})$, where $\mathcal{K}^{-1}\mathcal{T}$ is a compact operator. Hence, the operator $(I+\mathcal{K}^{-1}\mathcal{T})$ is invertible by the Fredholm theory.

## Asymptotics on imaginary axis

### Lemma 8.1

*i)* Let $g\in C_0^\infty$ satisfy the condition $|\hat{g}(k)|\le C|k|^N$ with some $N=2,3,...$, and $f\in L^2_{\beta }$ with $\beta >7/2$. Then

8.1 $$\begin{aligned} F(\mu ):=\left\langle \frac{\hat{g}}{\hat{D}(i\mu +0)}, \hat{f}\right\rangle =F(0)+\mu F'(0)+\cdots +\mu ^{N-2}\frac{F^{(N-2)}}{(N-2)!} +\mathcal{O}(|\mu |^{N-3/2}),~~ \mu \rightarrow 0,\nonumber \\ \end{aligned}$$

*ii)* Let $\rho$ satisfy (1.2)–(1.3), and $\hat{g}(k)$ is one of the functions $\frac{k_j^2\hat{\rho }(k)}{k^2}$, $\frac{k_j^2k_l^2\hat{\rho }(k)}{k^2}$, $k_j\nabla _j\hat{\rho }(k)$, $j, l=1,2$. Then

8.2 $$\begin{aligned} F_g(\mu ):=\left\langle \frac{\hat{\rho }}{\hat{D}(i\mu \!+\!0)}, \hat{g}\right\rangle =F_g(0)+\mu ^2\frac{F_g''(0)}{2} +\mu ^4\frac{F_g^{(4)}(0)}{4!}+\mathcal{O}(|\mu |^{\frac{11}{2}}),~~ \mu \rightarrow 0. \end{aligned}$$

*iii)* Let $\rho$ satisfy (1.2)–(1.3). Then

8.3 $$\begin{aligned} \varkappa (i\mu +0)=\left\langle \frac{\nabla \hat{\rho }}{\hat{D}(i\mu \!+\!0)}, \nabla \hat{\rho }\right\rangle =\varkappa (0)+\mu ^2\frac{\varkappa ''(0)}{2} +\mathcal{O}(|\mu |^{\frac{7}{2}}),~~ \mu \rightarrow 0. \end{aligned}$$

Formally, the expansions can be obtained by differentiation of $\hat{D}^{-1}(\lambda )$. The rigorous proof is given in [7]. The lemma implies asymptotics of $L^{-1}(i\mu +0)$ and $\Gamma _0(i\mu +0)$ for small real $\mu$.

### Lemma 8.2

(i) Let conditions (1.2)–(1.3) hold. Then
  8.4 $$\begin{aligned} L^{-1}(i\mu +0) =\begin{pmatrix} \frac{\mu ^{-2}}{a_{12}}-\frac{a_{14}}{a_{12}^{2}}+\mathcal{O}(|\mu |^{3/2}) &  0\\ 0&  \frac{\mu ^{-2}}{a_{22}}-\frac{a_{24}+v^2c_{24}}{a_{22}^{2}}+\mathcal{O}(|\mu |^{3/2})\end{pmatrix}\!,~~\mu \rightarrow 0,~~\mu \in \mathbb {R}, \end{aligned}$$
  where $$a_{j2}=-\frac{1}{2}q_j''(0)+m+u_j(0)>0$$, $$j=1,2$$.
(ii) Let in addition $(\Lambda _0,\Psi _0)\in \mathcal{F}_{\beta }$ with $\beta >7/2$. Then
  8.5 $$\begin{aligned} \Gamma _0(i\mu +0)=\Gamma _0(0)+\Gamma _0'(0)\mu +\Gamma _0''(0)\mu ^2+\Gamma _0'''(0)\mu ^3+\mathcal{O}(\mu ^{7/2}),\quad \mu \rightarrow 0,~~\mu \in \mathbb {R}. \end{aligned}$$

### Proof

(i) Formulas (7.13), (7.17) and (7.20), and asymptotics (8.2) imply
  8.6 $$\begin{aligned}  &   a_j(i\mu +0)=a_{j2}\mu ^2+a_{j4}\mu ^4+\mathcal{O}(|\mu |^{11/2}),\nonumber \\  &   p_2(i\mu +0)-p_2(0)=\frac{1}{2} p_2''(0) \mu ^2+\mathcal{O}(|\mu |^{4}), \quad \mu \rightarrow 0. \end{aligned}$$
  Hence, (8.4) with $c_{24}=\frac{(p''_{2}(0))^2}{4(I+\varkappa (0))}$ follows by (7.19) and (8.3).
(ii) To obtain (8.5), we substitute definition (7.7) of $K_0$ into (7.16) and apply Lemma 8.1. Consider, for example, the last summand in (7.16). One has
  8.7 $$\begin{aligned} \left\langle \frac{J\hat{\varrho }}{\hat{D}(\lambda )},\hat{K}_0\right\rangle = \left\langle \frac{J\hat{\varrho }}{\hat{D}(\lambda )}, i(v\cdot k)\hat{\Lambda }_0\right\rangle +\left\langle \frac{J\hat{\varrho }}{\hat{D}(\lambda )},\hat{\Psi }_0\right\rangle -\left\langle \frac{J\hat{\varrho }}{\hat{D}(\lambda )},\widehat{\mathcal{P}[r_0\rho ]}\right\rangle +\lambda \left\langle \frac{J\hat{\varrho }}{\hat{D}(\lambda )},\hat{\Lambda }_0\right\rangle \end{aligned}$$
  Evidently, for the first three summands in the RHS of (8.7) asymptotics (8.1) with $N=3$ hold. Similar asymptotics for the last summand follows from the equality $\langle \frac{J\hat{\varrho }}{\hat{D}(\lambda )},\hat{\Lambda }_0\rangle =\langle \frac{J\hat{\varrho }}{|k|\hat{D}(\lambda )},|k|\hat{\Lambda }_0\rangle .$ Finally, asympotics (8.5) for the last summand of (7.16) follows by (8.6).

Further, we obtain asymptotics of $L^{-1}(i\mu +0$ ) and $\Gamma _0(i\mu +0)$ for large $\mu$. In the case $v=(|v|,0)$, one has

$$ \hat{D}(\lambda ,k)=k^2+(i|v|k_1+\lambda )^2=\frac{1}{\gamma ^2}(k_1+i|v|\lambda \gamma ^2)^2+k_2^2+\gamma ^2\lambda ^2,\qquad \gamma :=1/\sqrt{1-v^2}. $$

Hence,

8.8 $$\begin{aligned} G(\lambda ,z)&=\frac{1}{2\pi }\int \frac{e^{-ikz}dk}{\hat{D}(\lambda ,k)} =\gamma e^{-|v|\lambda \gamma ^2 z_1}\int \frac{e^{-ik\tilde{z}}dk}{k_1^2+k_2^2+\gamma ^2\lambda ^2} \nonumber \\&=\gamma e^{-|v|\lambda \gamma ^2 z_1}R(-\gamma ^2\lambda ^2,\tilde{z}),~~ \tilde{z}=(\gamma z_1,z_2). \end{aligned}$$

Here $R(\zeta , x-y)$ is the integral kernel of the resolvent $\mathcal{R}(\zeta )=(-\Delta -\zeta )^{-1}$ of the 2D Laplacian. Recall that for $\mathcal{R}(\zeta )$ the following asymptotics hold [1, 4]: for $s,k=0,1,2,...$ and $|l|\le 2$,

8.9 $$\begin{aligned} \Vert \mathcal{R}^{(k)}(\zeta \pm i0)\Vert _{H^s_\sigma \rightarrow H^{s+l}_{-\sigma }} =\mathcal{O}(|\zeta |^{-\frac{1+k-l}{2}}), \quad \zeta \rightarrow \infty ,\quad \zeta>0,\quad \sigma >1/2+k. \end{aligned}$$

Let $\mathcal{G}(\lambda )$ be the integral operator with the integral kernel $G(\lambda ,x-y)$. Formula (8.8) and asymptotics (8.9) imply

8.10 $$\begin{aligned} \Vert \mathcal{G}^{(k)}(i\mu +0)\Vert _{H^s_\sigma \rightarrow H^{s+l}_{-\sigma }} =\mathcal{O}(|\mu |^{-1+l}), \quad \mu \rightarrow \infty ,\quad \mu \in \mathbb {R},\quad \sigma >1/2+k,\quad |l|\le 2. \end{aligned}$$

### Lemma 8.3

(i) Let conditions (1.2)–(1.3) hold. Then
  8.11 $$\begin{aligned} \partial ^{k} L^{-1}(i\mu +0)=\mathcal{O}(|\mu |^{-2-k}),\quad \mu \rightarrow \infty ,\quad \mu \in \mathbb {R},\quad k=0,1,2. \end{aligned}$$
(ii) Let in addition $(\Lambda _0,\Psi _0)\in \mathcal{F}_{\beta }$ with $\beta >7/2$. Then for $k=0,1,2$, and sufficient large $B>0$,
  8.12 $$\begin{aligned} |\Gamma _0^{(k)}(i\mu +0)| \le C|\mu |^{1-k}(1+\Vert (\Lambda _0,\Psi _0)\Vert _{\mathcal{F}_\beta }),\quad |\mu |\ge B,\quad \mu \in \mathbb {R}. \end{aligned}$$

### Proof

(i) Applying (8.10) with $s=l=0$, $k=0,1,2$ and $\sigma >1/2+k$, we obtain
  8.13 $$\begin{aligned} |\varkappa ^{(k)}(i\mu +0)|=|\langle \mathcal{D}^{(k)}(i\mu +0)\varrho ,\varrho \rangle | \le C\Vert \mathcal{D}^{(k)}(i\mu +0)\varrho \Vert _{H^{-1}_{-\sigma }}\Vert \varrho \Vert _{H^1_{\sigma }} \le C_1|\mu |^{-3}\Vert \varrho \Vert _{H^1_{\sigma }}^2,\quad \mu \rightarrow \infty . \end{aligned}$$
  For $p_j^{(k)}$, $u_j^{(k)}$, $q_j^{(k)}$ the similar bounds hold. Therefore, (7.20) implies
  8.14 $$\begin{aligned} a_j(i\mu +0)\sim m\mu ^2,\quad a_j'(i\mu +0)\sim 2m\mu ,\quad a_j''(i\mu +0)\sim 2m,\quad \mu \rightarrow \infty . \end{aligned}$$
  Hence, (8.11) follows.
(ii) By (8.10),
  $$ |\langle \mathcal{G}^{(k)}(i\mu +0)\Psi _0,\rho _1\rangle |\le C\Vert \mathcal{G}^{(k)}(i\mu +0)\Psi _0\Vert _{H^{-1}_{-\beta }}\Vert \rho _1\Vert _{H^1_{\beta }} \le C_1 |\mu |^{-2}\Vert \Psi _0\Vert _{L^{2}_{\beta }},~~ |\mu |\ge B,~~k=0,1,2. $$
  Here $\rho _1$ is one of the functions $\rho$, $\nabla \rho$, $y\rho$. The similar decay is valid for $\nabla \Lambda _0$ instead of $\Psi _0$. Moreover,
  $$ |\langle \mathcal{G}^{(k)}(i\mu +0)\Lambda _0,\rho _1\rangle | \le C\Vert \mathcal{G}^{(k)}\Lambda _0'\Vert _{H^{-2}_{-\beta }}\Vert \rho _2\Vert _{H^2_{\beta }} \le C_1 |\mu |^{-3}\Vert \nabla \Lambda _0\Vert _{L^{2}_{\beta }}. $$
  Here $\Lambda _0'=F^{-1}_{k\rightarrow x}[ |k|\hat{\Lambda }_0]$, $\rho _2(x)=F^{-1}_{k\rightarrow x}\frac{\hat{\rho }_1(k)}{|k|}$. Hence, (8.12) follows by (7.7) and (7.16).

### Lemma 8.4

Let either i) $v\ne 0$ and $I\gg 1$ or ii) $|v|\ll 1$ and condition (1.8) holds. Then

8.15 $$\begin{aligned} \textrm{det}\, L(i\mu +0)\ne 0~~ \textrm{for} ~~\mu \ne 0. \end{aligned}$$

We prove this lemma in Appendix B.

## Decay of the linearized dynamics

Here we prove time decay of the solution to the linearized equation under the symplectic orthogonality condition on the initial data.

### Definition 9.1

Denote by $\mathbf{\Pi }_{v}$ the symplectic orthogonal projection of $\mathcal{E}$ onto the tangent space $\mathcal{T}_{S(\sigma )}\mathcal{S}$, and $\textbf{P}_{v}:=\textbf{I}-\mathbf{\Pi }_{v}$. Denote by $\mathcal{Z}_{v}=\textbf{P}_{v}\mathcal{E}$ the space symplectic orthogonal to $\mathcal{T}_{S(\sigma )}\mathcal{S}$.

The tangent space $\mathcal{T}_{S(\sigma )}\mathcal{S}$ is invariant under the operator $\textbf{A}_{v}$ by Lemma 6.2, hence the space $\mathcal{Z}_{v}$ is also invariant.

### Proposition 9.2

Let conditions (1.2)–(1.3) and (8.15) hold, and let $X_0\in \mathcal{Z}_{v}\cap \mathcal{E}_\beta$ with $\beta >7/2$. Then $X(t)=e^{\textbf{A}_{v}t}X_0 \in C(\mathbb {R}, \mathcal{Z}_{v}\cap \mathcal{E}_{-\beta })$ and the decay holds

9.1 $$\begin{aligned} \Vert X(t)\Vert _{-\beta }\le C(\beta ,v)(1+t)^{-2}\Vert X_0\Vert _{\beta },\quad t>0. \end{aligned}$$

### Proof

The symplectic orthogonality conditions imply that

9.2 $$\begin{aligned} \Gamma _0(0)=0,\qquad \Gamma '_0(0)=0. \end{aligned}$$

We prove (9.2) in Appendix A. By (7.19),

$$ r(t)=\frac{1}{2\pi }\Big (\int e^{i\mu t} \zeta (\mu ) L^{-1}(i\mu +0)\Gamma _0(\mu +i0) d\mu +\!\int e^{i\mu t} (1-\zeta (\mu )) L^{-1}(i\mu +0) \Gamma _0(\mu +i0) d\mu \Big ). $$

Here $\zeta \in C_0^{\infty }(\mathbb {R})$, $\textrm{supp}\,\zeta \in (-2,2)$, $\zeta (\mu )=1$ for $|\mu | \le 1$. We apply double integration by parts to each term in the RHS separately. Then the decay (9.1) for *r*(*t*) follows by (8.4)–(8.5), (8.11)–(8.12) and (9.2). Due to (7.16), (7.19) and (8.14),

9.3 $$\begin{aligned} \tilde{r}(i\mu +0)=\displaystyle \frac{r_0}{\mu }+\mathcal{O}(|\mu |^{-2}),\quad \mu \rightarrow \infty . \end{aligned}$$

Hence, the third equation of (7.4) implies

$$ \tilde{\phi }(i\mu +0)=i\mu \tilde{r}-r_0=\mathcal{O}(|\mu |^{-1}),\quad \tilde{\phi }^{(k)}(i\mu +0)=\mathcal{O}(|\mu |^{-1-k}),\quad k=1,2, \quad \mu \rightarrow \infty , \quad \mu \in \mathbb {R}. $$

Moreover, $\hat{\phi }(i\mu +0)\in C^2(\mathbb {R})$. Hence double integration by parts gives

9.4 $$\begin{aligned} \phi (t)=\frac{1}{2\pi }\int e^{i\mu t} \tilde{\phi }(i\mu +0) d\mu =\mathcal{O}(t^{-2}), \quad t\rightarrow \infty . \end{aligned}$$

Similarly, definition (7.14) of $\tilde{\nu }$, asymptotics of type (8.13) for $\varkappa$ and $p_j$, asymptotics of type (8.12) for $\langle \frac{\hat{K}_0}{\hat{D}(\lambda )},J\hat{\varrho }\rangle$ and (9.3) imply that

9.5 $$\begin{aligned} \nu (t)=\frac{1}{2\pi }\int e^{i\mu t} \tilde{\nu }(i\mu +0) d\mu =\mathcal{O}(t^{-2}), \quad t\rightarrow \infty . \end{aligned}$$

Now we prove time decay for the field components. Denote $\mathcal{F}(t)=(\Lambda (t),\Psi (t))$ and rewrite the first two equations of (7.2) as

9.6 $$\begin{aligned} \dot{\mathcal{F}}(t)= \mathcal{B}_v \mathcal{F}(t)+\begin{bmatrix} 0\\ Q(t)\end{bmatrix},\quad \mathcal{B}_v =\begin{pmatrix} v\cdot \!\nabla &  1 \\ \Delta &  v\cdot \!\nabla \end{pmatrix},\quad \mathcal{Q}=\mathcal{P}[\rho \phi -\!v(r\cdot \nabla \rho )]-\nu J\varrho . \end{aligned}$$

Hence,

9.7 $$\begin{aligned} \mathcal{F}(t)=W_v(t)\mathcal{F}_0+\int _0^t W_v(t-s)\begin{bmatrix} 0\\ \mathcal{Q}(s)\end{bmatrix} ds,\quad t\ge 0, \end{aligned}$$

where $W_v(t)$ is the dynamical group of the equation $\dot{\mathcal{F}}(t)=\mathcal{B}_v\mathcal{F}(t).$ For the group $W_v(t)$ the dispersion decay holds [5, 7]:

9.8 $$\begin{aligned} \Vert W_v(t)\mathcal{F}_0\Vert _{\mathcal{F}_{-\beta }}\le C(1+t)^{-2}\Vert \mathcal{F}_0\Vert _{\mathcal{F}_\beta },\quad \beta >2,\quad |v|<1,\quad t\ge 0. \end{aligned}$$

Applying (9.8) to (9.7), and using (9.4)–(9.5), we obtain the decay (9.1) for the fields. Proposition 9.2 is proved.

## Symplectic decomposition of the dynamics

Now we choose $S(\sigma (t)):=\mathbf{\Pi } Y(t)$ in the splitting (5.1). This is possible for $t=0$ by assumption (4.1). Moreover, Lemma 3.4 implies that $S(\sigma (t))=\mathbf{\Pi } Y(t)$ and $Z(t)=Y(t)-S(\sigma (t))$ are well defined for $t\ge 0$ until $\Vert Z(t)\Vert _{-\beta } < z_{-\beta }(\tilde{v})$. We introduce the “exit time” $t_*$,

10.1 $$\begin{aligned} t_*=\sup \{t>0: \Vert Z(s)\Vert _{-\beta } < z_{-\beta }(\tilde{v}),~~0\le s\le t\}. \end{aligned}$$

Below we prove that $t_*=\infty$ if $d_\beta$ is sufficiently small. This would follow if we show that

10.2 $$\begin{aligned} \Vert Z(t)\Vert _{-\beta }<z_{-\beta }(\tilde{v})/2,~~~~~0\le t < t_*. \end{aligned}$$

For $0<t<t_*$, we fix the decomposition $Y(t)=S(\sigma (t))+Z(t)$ by setting $S(\sigma (t))=\mathbf{\Pi } Y(t)$ which is equivalent to the symplectic orthogonality condition:

10.3 $$\begin{aligned} \Omega (Z(t),\tau _j(t))=0,\quad \tau _j(t)=\tau _j(\sigma (t)),\quad j=1,\dots ,4, ~~~~~~~0\le t<t_*. \end{aligned}$$

We will use parameter $c(t)=b(t)-\displaystyle \int ^t_0 v(\tau )d\tau$ instead of parameter *b*. One has

10.4 $$\begin{aligned} \dot{c}(t)=\dot{b}(t)-v(t)=u(t)-v(t), \quad 0\le t<t_*. \end{aligned}$$

### Lemma 10.1

Let $Y(t)=S(\sigma (t))+Z(t)$ be a solution to (1.6) and condition (10.3) be satisfied. Then

10.5 $$\begin{aligned} \dot{\varpi }(t):=(\dot{c}(t), \,\dot{v}(t)) =\mathcal{O}(\Vert Z\Vert _{-\beta }^2), ~~~~~~~0\le t<t_*, \end{aligned}$$

uniformly in *v* and *Z* with $\Vert Z\Vert _{-\beta }\le z_{-\beta }(\tilde{v})$ and $|v|<\tilde{v}<1$.

### Proof

Differentiating the orthogonality conditions (10.3) with respect to *t* gives

10.6 $$\begin{aligned} 0=\Omega (\dot{Z},\tau _j)+\Omega (Z,\dot{\tau }_j)=\Omega (\textbf{A}_{u,v}Z+T_{u,v}+N(\sigma ,Z),\tau _j)+\Omega (Z,\dot{\tau }_j), ~~~~~~~0\le t<t_*. \end{aligned}$$

By (3.7) and (5.18), $T_{u,v}(t)=-\sum \limits _{l=1}^2[\dot{c}_l\tau _l+\dot{v}_l\tau _{l+2}]$. Hence, $\Omega (T,\tau )=\mathbf{\Omega }\dot{\varpi }$. Further, (6.1) and (10.3) imply

$$\begin{aligned}  &   \Omega (\textbf{A}_{u,v}Z,\tau _j)=-\Omega (Z,\textbf{A}_{u,v}\tau _j)=-\Omega (Z,\dot{c}\cdot \nabla \tau _j),\\  &   \Omega (\textbf{A}_{u,v}Z,\tau _{j+2})=-\Omega (Z,\textbf{A}_{u,v}\tau _{j+2})=-\Omega (Z,\dot{c}\cdot \nabla \tau _{j+2}),\quad j=1,2, \end{aligned}$$

Finally, $\Omega (Z,\dot{\tau }_j)= \Omega (Z,\dot{v}\cdot \nabla _v\tau _j)$, $j=1,...,4$. As the result, (10.6) becomes

10.7 $$\begin{aligned} 0=\mathbf{\Omega }\dot{\varpi }+\mathcal{M}_0(\sigma ,Z)\dot{\varpi }+\mathcal{N}_0(\sigma ,Z). \end{aligned}$$

Here the matrix $\mathbf{\Omega }$ is invertible by Lemma 3.3, $\mathcal{M}_0(\sigma ,Z)=\mathcal{O}(\Vert Z\Vert _{-\beta })$, and $\mathcal{N}_0(\sigma ,Z)=\mathcal{O}\big (\Vert Z\Vert _{-\beta }^2\big )$ uniformly in $\sigma \in \Sigma (\tilde{v})$ and $\Vert Z\Vert _{-\beta }<z_{-\beta }(\tilde{v})$. Then (10.5) follows, since $\Vert Z\Vert _{-\beta }$ is small.

Now we rewrite (5.16) as

10.8 $$\begin{aligned} \dot{Z}(t)=\textbf{A}_{u(t),v(t)}(t)Z(t)+\tilde{N}(t), \quad \tilde{N}(t):=T_{u(t),v(t)}+N(\sigma , Z(t)), \quad 0\le t<t_*. \end{aligned}$$

where

10.9 $$\begin{aligned} \Vert \tilde{N}(t)\Vert _{\beta }\le C(\tilde{v})\Vert Z\Vert _{-\beta }^2,\qquad 0\le t<t_*. \end{aligned}$$

uniformly in *v* and *Z* with $\Vert Z\Vert _{-\beta }\le z_{-\beta }(\tilde{v})$ and $|v|<\tilde{v}<1$.

## Frozen transversal dynamics

We fix an arbitrary $t_1\in [0,t_*)$ and denote $\textbf{A}_1=\textbf{A}_{v_1,v_1}$, where $v_1:=v(t_1)$. Then

11.1 $$\begin{aligned} \dot{Z}(t)=\textbf{A}_1Z(t)+(\textbf{A}_{u(t),v(t)}(t)-\textbf{A}_1))Z(t)+\tilde{N}(t),\qquad 0\le t<t_*. \end{aligned}$$

The upper left corner of the matrix $\textbf{A}_{u(t),v(t)}(t)-\textbf{A}_1$ contains the “bad terms” $[u(t)\!-\!v(t_1)]\cdot \nabla$. To kill them we change the variables $(y,t)\mapsto (y_1,t)=(y+a_1(t),t)$, where we denote

11.2 $$\begin{aligned} a_1(t):=\int _{t_1}^t(u(s)-v(t_1))ds, ~~~~0\le t\le t_1. \end{aligned}$$

Precisely, we define $\Lambda ^+(y_1,t):=\Lambda (y,t)=\Lambda (y_1-a_1(t),t)$, $\Psi ^+(y_1,t):=\Psi (y,t)=\Psi (y_1-a_1(t),t))$, and obtain the final form of the “frozen equation” for the transversal dynamics:

11.3 $$\begin{aligned} \dot{Z}^+(t)=\textbf{A}_1 Z^+(t)+\textbf{B}(t) Z^+(t)+\tilde{N}^+(t),\quad Z^+(t)\!:=(\Lambda ^+(t),\Psi ^+(t),r(t),\pi (t)), \quad 0\le t\le t_1. \end{aligned}$$

Here $\tilde{N}^+(t)$ is expressed in terms of $y=y_1-a_1(t)$, $$ \textbf{B}(t)=\begin{pmatrix} 0 &  \textbf{B}_{1}(t)\\ \textbf{B}_{2}(t) &  \textbf{B}_{3}(t) \end{pmatrix}, ~\textbf{B}_j=\begin{pmatrix} 0 &  0\\ B_{j}(t) &  0 \end{pmatrix},$$ where

$$\begin{aligned} B_{1}(t)\Lambda= &   \langle (v_1-v(t))\cdot \Lambda ,\nabla \rho \rangle +\frac{1}{I}\langle \Lambda ,J\varrho \rangle (PJ(v(t)-v_1)),\\ B_{2}(t)r= &   \mathcal{P}[(v_1-v(t))r\cdot \nabla \rho )]+\frac{1}{I}(r\cdot PJ(v(t)-v_1))J\varrho ,\\ B_{3}(t)r= &   (Q_1-Q(t))r+\frac{1}{I}\big ((r\cdot PJv_1)PJv_1-(r\cdot PJv(t))PJv(t)\big ). \end{aligned}$$

Denote $\overline{a}_1(s):=\sup _{0\le t\le s} |a_1(t)|$, $0\le s\le t_1$ and reduce the exit time:

11.4 $$\begin{aligned} t_*'=\sup \{t\in [0,t_*): \overline{a}_1(s)\le 1,~~0\le s\le t\}. \end{aligned}$$

Using (5.3) and (10.5), we obtain

11.5 $$\begin{aligned} \Vert \textbf{B}(t)Z^+(t)\Vert _{\beta }\le C(\tilde{v})\Vert Z(t)\Vert _{-\beta }|v(t)-v_1| \le C(\tilde{v})\Vert Z(t)\Vert _{-\beta }\int _{t_1}^{t} \Vert Z(s)\Vert _{-\beta }^2 ds, \quad 0\le t\le t_1<t_*'. \end{aligned}$$

Similarly,

11.6 $$\begin{aligned} \Vert \tilde{N}^+(t)\Vert _{\beta }\le C(\tilde{v}) \Vert Z(t)\Vert _{-\beta }^2, \quad 0\le t\le t_1<t_*'. 
\end{aligned}$$

## Decay of the transversal dynamics

### Theorem 12.1

Let all conditions of either Theorem 4.1 or Theorem 4.2 be satisfied. Then $t_*=\infty$, and

12.7 $$\begin{aligned} \Vert Z(t)\Vert _{-\beta }\le C(\tilde{v},d_\beta )(1+t)^{-2},\qquad t\ge 0. \end{aligned}$$

### Proof

*Step i)* Equation (11.3) implies

$$ \textbf{P}_1Z^+(t)=e^{\textbf{A}_1t}\textbf{P}_1Z^+(0)+\int _0^te^{A_1(t-s)}  \textbf{P}_1\big (\textbf{B}(s)Z^+(s)+\tilde{N}^+(s)\big )ds. $$

Applying Proposition 9.2 and (11.5)–(11.6), we obtain

12.8 $$\begin{aligned} \Vert \textbf{P}_1Z^+(t)\Vert _{-\beta }&\!\!\le \!\!&C\Big (\frac{\Vert \textbf{P}_1Z^+(0)\Vert _{\beta }}{(1+t)^2}+\int _0^t\frac{\Vert \textbf{P}_1[\textbf{B}(s)Z^+(s)+\tilde{N}^+(s)]\Vert _{\beta }}{(1+t-s)^2}ds\Big )\nonumber \\&\!\!\le \!\!&C_1\Big (\frac{\Vert Z(0)\Vert _{\beta }}{(1+t)^2} +\int _0^t\frac{\Vert Z(s)\Vert _{-\beta }}{(1\!+t-\!s)^2}\left[ \int _s^{t_1}\Vert Z(\tau )\Vert _{-\beta }^2 d\tau +\Vert Z(s)\Vert _{-\beta }\right] ds\Big )\nonumber \\ \end{aligned}$$

for $t_1<t_*'$ and $0\le t\le t_1$. Now we introduce the “majorant”

12.9 $$\begin{aligned} \mathcal{M}(t):= \sup _{s\in [0,t]}(1+s)^2\Vert Z(s)\Vert _{-\beta } \end{aligned}$$

and reduce the exit time once more. Let $\varepsilon$ be a fixed positive number which we will specify below. Denote

12.10 $$\begin{aligned} t_*''=\sup \{t\in [0,t_*'): \mathcal{M}(s)\le \varepsilon ,~~0\le s\le t\}. \end{aligned}$$

### Lemma 12.2

For sufficiently small $\varepsilon >0$ and $t_1<t_*''$ the bound holds

12.11 $$\begin{aligned} \Vert Z(t)\Vert _{-\beta }\le C\Vert \textbf{P}_1Z^+(t)\Vert _{-\beta },\quad 0\le t \le t_1, \end{aligned}$$

where *C* depends only on $\rho$ and $\tilde{v}$.

The proof is similar to that of Lemma 9.2 in [2]. Lemma 12.2 together with (12.8) imply

$$ \Vert Z(t)\Vert _{-\beta } \le C\Big (\frac{\Vert Z(0)\Vert _{\beta }}{(1+t)^2}+\int \limits _0^t\frac{\Vert Z(s)\Vert _{-\beta }}{(1+t-\!s)^2} \Big [\int \limits _s^{t_1}\Vert Z(\tau )\Vert _{-\beta }^2 d\tau +\Vert Z(s)\Vert _{-\beta }\Big ]ds\Big ),~~t_1<t_*'. $$

Multiplying both sides by $(1+t)^2$, and taking the supremum in $t\in [0,t_1]$, we get

$$ \mathcal{M}(t_1) \le C\Big (\Vert Z(0)\Vert _{\beta }+\! \sup _{t\in [0,t_1]}\int \limits _0^t \frac{(1+t)^2}{(1+t-\!s)^2}\Big [\frac{\mathcal{M}(s)}{(1+s)^{2}} \int \limits _s^{t_1}\frac{\mathcal{M}^2(\tau )d\tau }{(1+\tau )^{4}}+\frac{\mathcal{M}^2(s)}{(1+s)^{4}}\Big ]ds\Big ),~~t_1<t_*'. $$

Taking into account that *m*(*t*) is a monotone increasing function, we get

12.12 $$\begin{aligned} \mathcal{M}(t_1)\le &   C\Vert Z(0)\Vert _{\beta }+C_1\big (\mathcal{M}^3(t_1)+\mathcal{M}^2(t_1)\big ),~~~~t_1<t_*'. \end{aligned}$$

This inequality and assumption (4.1) imply that $\mathcal{M}(t_1)$ is bounded for $t_1<t_*''$, and moreover,

12.13 $$\begin{aligned} \mathcal{M}(t_1)\le C_2\Vert Z(0)\Vert _{\beta },~~~~~~~~~t_1<t_*''\,. \end{aligned}$$

*Step ii)* We choose $d_{\beta }$ in (4.1) so small that $\Vert Z(0)\Vert _{\beta }<\varepsilon /(2C_2)$. Then (12.10) implies that $t''_*=t'_*$ and (12.13) holds for all $t_1<t'_*$. By (10.4),

$$ u(s)-v(t_{1})=u(s)-v(s)+v(s)-v(t_{1})=\dot{c}(s)+\displaystyle \int _s^{t_1}\dot{v}(\tau )d\tau . $$

Hence, Lemma 10.1 and (11.2) imply that

$$\begin{aligned} |a_{1}(t)|\!\!\le &   \!\! \int _{t}^{t_{1}}\left( |\dot{c}(s)|+\int _{s}^{t_{1}}|\dot{v}(\tau )|d\tau \right) ds\\ \!\!\le &   \!\! C\mathcal{M}^2(t_1)\int _{t}^{t_{1}}\left( \frac{1}{(1+s)^4}+\int _{s}^{t_{1}}\frac{d\tau }{1+\tau )^4}\right) ds\le C_1\mathcal{M}^2(t_1)\le C_2\varepsilon ^2,\quad t<t_*'. \end{aligned}$$

We choose $\varepsilon$ so small that $C_2\varepsilon ^2<1$. Then $t'_*=t_*$ by (11.4). Now (12.13) holds for all $t_1<t_*$. Hence, (10.2) also holds if $\Vert Z(0)\Vert _{\beta }$ is sufficiently small. Finally, this implies that $t_*=\infty$.

## Soliton asymptotics

Here we prove Theorems 4.1 and 4.2.

**Asymptotics for the vector components**. By (5.1), (5.8), (5.19) and (10.4),

13.1 $$\begin{aligned} \dot{q}(t)=\dot{b}(t)+\dot{r}(t)=v(t)+\dot{c}(t)+\frac{1}{m}(\pi (t)-\!\langle \Lambda (t),\rho \rangle )+N_3(t)=v(t)+\dot{c}(t)+\mathcal{O}(\Vert Z(t)\Vert _{-\beta }).\nonumber \\ \end{aligned}$$

Further, (10.5) and (12.7) imply that $|\dot{c}(t)|+|\dot{v}(t)|\le C(\tilde{v},d_\beta )(1+t)^{-4}$ for $t\ge 0$. Therefore,

13.2 $$\begin{aligned} c(t)=c_+ +\mathcal{O}(t^{-3}),\quad v(t)=v_+ +\mathcal{O}(t^{-3}),\quad t\rightarrow \infty . \end{aligned}$$

Hence, (12.7) and (13.1)–(13.2) imply that

13.3 $$\begin{aligned} \dot{q}(t)=v_++\mathcal{O}(t^{-2}),\quad b(t)=c(t)+\displaystyle \int _0^tv(s)ds=v_+t+a_++\mathcal{O}(t^{-2}),\quad t\rightarrow \infty . \end{aligned}$$

Hence, asymptotics (4.2) for *q*(*t*) follows by (5.1) and (12.7). Finally, (1.5), (5.1) and (12.7) imply that

13.4 $$\begin{aligned} \omega (t)= &   \frac{1}{I}\langle A(x,t),J\varrho (x\!-\!q(t)\rangle =\frac{1}{I}\langle A_{v(t)}(x)+\Lambda (x,t),J(\varrho (x\!+\!r(t))-\varrho (x))\rangle \nonumber \\  &   +\frac{1}{I}\langle \Lambda (x,t),J\varrho (x)\rangle = \mathcal{O}(t^{-2}),\quad t\rightarrow \infty . \end{aligned}$$

**Asymptotics for the fields**. For the field part of the solution, $F(x,t)=(A(x, t), \Pi (x, t))$, we define the accompanying soliton field as $F_{\textrm{v}(t)}(x,t) = \big (A_{\textrm{v}(t)}(x-q(t)), \Pi _{\textrm{v}(t)}(t)(x-q(t))\big )$, where $\textrm{v}(t) = \dot{q} (t)$. Denote $Z(t)\! =(\Lambda (t),\Psi (t))\!= F(t) -F_{\textrm{v}(t)}(t)$. Substitution into the first two equations of (1.6) gives

13.5 $$\begin{aligned} \dot{Z}(t)=\begin{pmatrix} 0&  1\\ \Delta & 0 \end{pmatrix}Z(t)-R(t),\quad R(t):=\dot{\textrm{v}}(t)\cdot \nabla _{\textrm{v}}F_{\textrm{v}(t)}(x-q(t))+\omega (t)\begin{pmatrix} 0\\ J\varrho (x-q(t)) \end{pmatrix}. \end{aligned}$$

Then

13.6 $$\begin{aligned} Z(t)=W_0(t)\Big (Z(0)-\int _0^{\infty }W_0(-s)R(s)ds\Big )+\int _t^{\infty }W_0(t-s)R(s)ds=W_0(t)\Psi _++r_+(t). \end{aligned}$$

The last two equations of (1.6) together with (5.1), (12.7) and (13.3) imply

$$\begin{aligned} m\dot{\textrm{v}}(t)= &   m\ddot{q}(t)=-\langle A(x,t)\cdot \dot{q}(t),\nabla \rho (x\!-\!q(t))\rangle +\omega (t) \langle \nabla \cdot JA(x,t), \varrho (x\!-\!q(t))\rangle \\  &   -\langle \Pi (x,t),\rho (x\!-\!q(t))\rangle +\langle A(x,t),\dot{q}(t)\cdot \nabla \rho (x\!-\!q(t))\rangle \\= &   J\dot{q}(t)\langle A(x,t),J\nabla \rho (x\!-\!q(t))\rangle +\omega (t) \langle \nabla \cdot JA(x,t), \varrho (x\!-\!q(t))\rangle -\langle \Pi (x,t),\rho (x\!-\!q(t))\rangle \\= &   J\dot{q}(t)\langle ( A_{v(t)}(x)+\Lambda (x,t)),J\nabla (\rho (x+r(t))-\rho (x))\rangle +J\dot{q}(t)\langle \Lambda (x,t)),J\nabla \rho (x))\rangle \\  &   -\langle \Pi _v(x)+\!\Psi (x,t),\rho (x\!+\!r(t))\!-\!\rho (x)\rangle -\langle \Psi (x,t),\rho (x)\rangle \\  &   + \omega (t) \langle \nabla \cdot \! JA(x,t), \varrho (x\!-\!q(t))\rangle =\mathcal{O}(t^{-2}),\quad t\rightarrow \infty . \end{aligned}$$

Hence, $\Vert R(t)\Vert _\mathcal{F} = \mathcal{O}(t^{-2})$ by (13.5) and (13.4). Therefore $\Psi _{+} =Z(0)-\int _0^{\infty }W_0(-s)R(s)ds\in \mathcal{F}$ and $\Vert r_{+}(t)\Vert _\mathcal{F}= \mathcal{O}(t^{-1})$ due to the unitarity of $W_0(t)$.

$$\square $$

## Symplectic orthogonality conditions

Here we prove (9.2). In the case $v=(|v|,0)$, (3.3) and (3.7) imply

$$\begin{aligned}  &   -\Omega (X_0,\tau _j)=\langle \Lambda _0,\partial _j \Pi _{v}\rangle -\langle \Psi _0,\partial _j A_{v}\rangle +\pi _{0j}\\  &   \quad =\!\int (\hat{\Lambda }_0\cdot v) \frac{k_j(v\cdot k)\rho }{\hat{D}_0}dk -i\!\int (\hat{\Psi }_0\cdot v)\frac{k_j\rho }{\hat{D}_0} dk +\pi _{0j}\\  &   \quad =v^2\int \hat{\Lambda }_{01} \frac{k_1k_j\rho }{\hat{D}_0}dk -i|v|\int \hat{\Psi }_{01}\frac{k_j\rho }{\hat{D}_0} dk +\pi _{0j}=0,\quad j=1,2 \end{aligned}$$

since $\Lambda _0\cdot k=0$ and $\Psi _0\cdot k=0$. Hence, by (7.16),

$$ \Gamma _{0j}(0)=\pi _{0j} -i|v|\langle \frac{\hat{K}_{01}(0)}{\hat{D}_0},k_j\hat{\rho }\rangle =\pi _{0j}+v^2\int \frac{k_1k_j\hat{\rho }\hat{\Lambda }_{01}}{\hat{D}_0} dk-i|v|\int \frac{\hat{\rho }\hat{\Psi }_{01}k_j}{\hat{D}_0}dk=0. $$

Further, (3.7) implies

A.1 $$\begin{aligned} 0=\Omega (X_0,\tau _{j+2})=\langle \Lambda _0, \partial _{v_j} \Pi _v\rangle -\langle \Psi _0,\partial _{v_j} A_{v}\rangle +r_0\cdot (me_j+\langle \partial _{v_j} A_{v},\rho \rangle ), \quad j=1,2, \end{aligned}$$

where

$$ \partial _{v_j}A_{v}=\displaystyle \frac{\hat{\rho }}{\hat{D}_0}\Big (e_j+\displaystyle \frac{2(v\cdot k)k_j}{\hat{D}_0}v-\displaystyle \frac{k_j(k^2+(v\cdot k)^2)}{k^2\hat{D}_0}k\Big ),\quad \partial _{v_j}\Pi _{v}= ik_jA_v+i(v\cdot k)\partial _{v_j} A_v $$

by (3.3). In the case $v=(|v|,0)$, (A.1) leads to

$$\begin{aligned}  &   -i|v|\int \frac{\hat{\Lambda }_{01}(k^2+v^2k_1^2)\rho }{\hat{D}_0^2}k dk-i|v|\int \frac{k_1\rho }{\hat{D}_0}\Lambda _{0} dk -2v^2\!\int \frac{ \hat{\Psi }_{01}k_1\rho }{\hat{D}_0^2}kdk-\int \frac{\rho }{\hat{D}_0}\Psi _{0} dk\\  &   \quad +m r_{0}+r_{01}e_1\int \frac{(v^2k_1^2+k^2)k_2^2|\hat{\rho }|^2}{k^2\hat{D}_0^2}dk -r_{02}e_2\int \frac{(v^2k_2^2+k^2(v^2-1))k_1^2|\hat{\rho }|^2}{k^2\hat{D}_0^2}dk=0. \end{aligned}$$

On the other hand, (7.16) implies

$$\begin{aligned} \Gamma _0'(0)= &   -i|v|\left\langle \frac{(k^2+v^2k_1^2)\Lambda _{01}}{\hat{D}^2_0},k\hat{\rho }\right\rangle -2v^2\left\langle \frac{k_1\Psi _{01}}{\hat{D}^2_0},k\hat{\rho }\right\rangle +2v^2\left\langle \frac{k_1k_2(r_0\cdot Jk)\hat{\rho }}{k^2\hat{D}^2_0},k\hat{\rho }\right\rangle \\  &   -i|v|\left\langle \frac{k_1\Lambda _0}{\hat{D}_0},\hat{\rho }\right\rangle -\left\langle \frac{\Psi _0}{\hat{D}_0},\hat{\rho }\right\rangle +\left\langle \frac{\hat{\rho }(r_0\cdot Jk)}{k^2\hat{D}_0}Jk,\hat{\rho }\right\rangle +mr_0=0 \end{aligned}$$

since

$$\begin{aligned} 2v^2\left\langle \frac{k_1k_2(r_0\cdot Jk)\hat{\rho }}{k^2\hat{D}_0^2},k\hat{\rho }\right\rangle +\left\langle \frac{\hat{\rho }(r_0\cdot Jk)}{k^2\hat{D}_0}Jk,\hat{\rho }\right\rangle= &   r_{01}e_1\int \frac{(v^2k_1^2+k^2)k_2^2|\hat{\rho }|^2}{k^2\hat{D}_0^2}\\  &   -r_{02}e_2\int \frac{(v^2k_2^2+k^2(v^2-1))k_1^2|\hat{\rho }|^2}{k^2\hat{D}_0^2}dk. \end{aligned}$$

## Regularity in continuous spectrum

*(i)* First, we consider the case $v\ne 0$ and prove that (8.15) holds for large $I\gg 1$. We start with an auxiliary lemma.

### Lemma B.1

Let $0<|v|<1$ and *h*(*k*) be one of the functions $|\nabla \hat{\rho }(k)|^2$, $\displaystyle \frac{|\hat{\rho }(k)|^2 k_j^2}{k^2}$, $\displaystyle \frac{|\hat{\rho }(k)|^2 k_j^2k_\ell ^2}{k^2}$, where $j,\ell =1.2$. Then

$$ \mathrm{Im{\hspace{0.5mm}}}\Big (\int \frac{h(k)\,dk}{\hat{D}(i\mu +0)}\Big )<0, \quad \mu \ne 0. $$

### Proof

We take $v=(|v|,0)$. Then $\hat{D}(i\mu +0)=\hat{D}(v,k,i\mu +0)=k^2+(i|v|k_1+(i\mu +0))^2$. Denote $K_v(\mu )=\{k\in \mathbb {R}^2: (k_1-\mu |v|\gamma ^2)^2+k_2^2\gamma ^2=\mu ^2\gamma ^4\}$, $\gamma =1/\sqrt{1-v^2}$. By the Plemelj formula,

$$ \mathrm{Im{\hspace{0.5mm}}}\Big (\int \frac{h(k)\,dk}{\hat{D}(i\mu +0)}\Big )=-\pi \int _{K_v(\mu )} \frac{h(k)}{|\nabla _k\hat{D}(i\mu +0)|}dS, $$

where $h(k)\ge 0$ on $K_v(\mu )$. Hence, it suffices to show that $h(k) \not \!\!\equiv \, 0$ on $K_v(\mu )$ for $\mu \ne 0$. Assume that, on the contrary, $h(k) \equiv 0$ on $K_v(\mu )$. Then $\hat{\rho }(k) \equiv 0$ in the ring $|\mu |(1-|v|)\gamma ^2 \le |k|\le |\mu |(1+|v|)\gamma ^2$ by the symmetry (1.2) of $\hat{\rho }(k)$. Hence, $\hat{\rho }(k) \equiv 0$ in $\mathbb {C}^2$ due to the analyticity of $\hat{\rho }(k)$, in contradiction to the last assumption in (1.2).

The lemma and definitions (7.17) and (7.20) imply that $\mathrm{Im{\hspace{0.5mm}}}a_j(i\mu +0)>0$ for $\mu \ne 0$. Thus, in the case $v\ne 0$ and $I=\infty$, $\textrm{det}\, L(i\mu +0)=a_1(i\mu +0)a_2(i\mu +0)\ne 0$ by (7.19). Therefore $\textrm{det}\, L(i\mu +0)\ne 0$ for large *I* by the continuity of $\textrm{det}\, L(i\mu +0)$.

*(ii)* In the case $v=0$, one has

B.1 $$\begin{aligned} \textrm{det}\,L(i\mu +0)=a_1(i\mu +0)a_2(i\mu +0)=\mu ^4\Big (m+\int \frac{|\hat{\rho }(k)|^2\,dk}{2(k^2+(i\mu +0)^2)}\Big )^2. \end{aligned}$$

Using the Plemelj formula and the Wiener condition (1.8), we get

$$ \mathrm{Im{\hspace{0.5mm}}}\Big (\int \frac{|\hat{\rho }(k)|^2\, dk}{k^2+(i\mu +0)^2}\Big )=-\pi \int _{K_0(\mu )} \frac{|\hat{\rho }(k)|^2}{\sqrt{2} |\mu |}dS<0, \quad K_0(\mu )=\{k\in \mathbb {R}^2: k^2=\mu ^2\}. $$

Hence, $\textrm{det}\, L(i\mu +0)\ne 0$ for $\mu \ne 0$ in the case $v=0$. Therefore $\textrm{det}\, L(i\mu +0)\ne 0$ for small *v* by the continuity of $\textrm{det}\, L(i\mu +0)$.
